# Immersion in the search for effective cancer immunotherapies

**DOI:** 10.1186/s10020-021-00321-3

**Published:** 2021-06-16

**Authors:** Steven A. Rosenberg

**Affiliations:** National Cancer Institute, National Institutes of Health, Bethesda, MD USA

**Keywords:** Cancer, Immunotherapy, T-cells, Adoptive transfer, Lymphocytes

The unique clinical course of patients can provide physicians with insights not found in textbooks. Such was the case with two patients that I encountered during my surgical residency training at the Peter Bent Brigham Hospital in Boston. I was assigned to the West Roxbury VA Hospital in the summer of 1968 when I encountered a 63-year-old veteran complaining of right upper quadrant abdominal pain. The diagnosis appeared to be gall bladder inflammation caused by gallstones and surgical removal of the gall bladder was indicated. As I reviewed the patient’s chart, a remarkable story unfolded.

Twelve years earlier, surgeons at the V.A. Hospital had resected two-thirds of the patient’s stomach that contained a large invasive gastric cancer. The operative note revealed that at least three tumors had spread to his liver as well as multiple lymph nodes and these could not be removed. Biopsies sent to the pathologist confirmed the diagnosis of cancer. No further treatment was given. The patient was seen again shortly after discharge from the hospital and was recovering normally. Five months later, he returned to the hospital, had gained weight and had returned to work. Now, 12 years later, I operated to remove his gall bladder and there was no remaining trace of the cancer. Somehow this cancer had undergone complete regression in the absence of any treatment, one of the rarest events in medicine. Only a few cases had ever been reported of a stomach cancer that underwent this kind of regression, and I eventually published this case (Rosenberg et al. 1972). It seemed likely to me that the patient’s immune system had recognized his cancer as foreign and destroyed it.

I then began an experiment naïve in its simplicity. I wanted to transfuse the patient’s blood into another cancer patient to see if his circulating immune, lymphocytes could impact another patient’s cancer. I identified a patient with advanced stomach cancer in the hospital whose blood type matched our patient, and I received permission to transfuse him with the blood of the patient who had experienced the spontaneous cancer regression. The second patient showed no improvement and died shortly thereafter from his growing malignancy. But the seed of using the patient’s own immune lymphocytes for therapy had been planted.

The unusual case of a second patient also intrigued me. A few years earlier, a patient at the Brigham Hospital had received a transplanted kidney from a person killed in a car accident. The patient who received the kidney transplant soon developed metastatic cancer that was traced to the transplanted kidney which had unknowingly harbored a cancer. The patient was receiving immunosuppressive drugs to prevent the kidney transplant from rejecting. When the immunosuppressive medications were stopped, the kidney was rejected and needed to be removed. The cancer deposits at distant sites then disappeared as well. This patient’s course strongly implied that an immune reaction was capable of rejecting large, vascularized metastatic cancers if the immune stimulus was strong enough, in this case the rejection of foreign tissue from another individual.

These two patients thus suggested a path of study to attempt to guide the immune system to destroy growing cancers, a journey that occupied me ongoing for the next 40 years.

## Early education

My career path changed when I was about 6 years old from wanting to be a cowboy to an intense, almost spiritual desire, to become a physician. Stories I heard from my parents were likely at the root of this decision.

My mother and father were born in small towns in Poland. Their families lost everything in the pogroms against the Jews before World War I, and they spent the war hiding in cellars, living off the kindness of strangers until they separately came to America in the early 1920s. They married in 1927 and I was born in 1940 when we were living in an apartment in the Bronx. I remember the months after World War II when my parents tried to reach relatives who had stayed in Poland, only to slowly learn about so many that had died in the death camps. My parents had experienced evils that I could hardly understand. As a child, I wanted to stop their suffering, stop everyone’s suffering.

I believe that this family history ultimately influenced my choosing to work on cancer. Cancer randomly attacks people of all ages and forces its victims and their families to watch impotently as it grows and spreads. Cancer murders innocents. It is a holocaust.

Neither my father nor mother had much time for education and my older brother and sister guided my education. They inundated me with books on every subject, and I remember being especially thrilled by one that taught me that cells could be kept alive outside the body. I was thrilled by books such as Microbe Hunters by Paul de Kruif, The Brothers Mayo by Helen Clapesattle, Arrowsmith by Sinclair Lewis, and The Cry and the Covenant by Morton Thompson. My brother became a surgeon and over many discussions flamed my interest in laboratory research.

My interest in medicine and science led me to the Bronx High School of Science and to Johns Hopkins for college and medical school and to a surgical residency in Boston in 1963. I knew that I wanted to care for patients but also do research aimed at developing new treatments. I realized that innovative research required a deeper background in basic science and after 1 year as a surgical intern, I received permission to leave the residency and spent 4 years completing a Ph.D. in Biophysics at Harvard. My first 2 years were in classes in physics, advanced mathematics, physical chemistry, and genetics followed by 2 years in laboratory research studying the protein structure of human cell membranes (Rosenberg and Guidotti 1968, 1969). I wanted a deep background in science so that I could draw upon disparate areas of scientific knowledge and develop confidence that with effort and a good book I could understand any aspect of science. I did not want to be intimidated by what I did not know.

I returned to the Brigham residency but stayed for only 1 year and then took another leave of absence to do research in the laboratory of Harvard Immunologist, John David. After 1 year there (1970) with the war in Vietnam raging, I joined the Public Health Service and became an Immunology Fellow at the National Cancer Institute in Bethesda, Maryland. The Immunology Branch was led by John Fahey and then by William Terry who gave me the freedom to pursue my interests in trying to identify cancer antigens on the membranes of cancer cells. I studied sialic acid on the cell membrane and its influence on immune reactions (Rosenberg and Einstein 1972), but the 2 years I spent at the NCI was too short a time to make substantial progress. I knew I wanted to do research that I could apply to patients and therefore needed additional clinical training and so I returned to Boston to finish the surgical residency.

In the last months of my residency, Francis Moore, Chief of Surgery at the Brigham Hospital, offered me the job as Chief of Surgery at Harvard’s new Dana Farber Cancer Center and a formal appointment at Harvard. Before agreeing to the Dana Farber offer, I was interviewed by Nathaniel Berlin, the National Cancer Institute Scientific Director. Several months later, he called and told me that the current Chief of Surgery at the National Cancer Institute was resigning, and he offered me the position. My choice was clear and the day after completing the surgical residency on June 30, 1974, I returned to Bethesda as the Chief of the Surgery Branch in the National Cancer Institute in charge of not only their clinical programs but a large resource to do laboratory research.

## Early research at the NIH

When I came to the NIH July 1, 1974, I was determined to work on understanding whether there was any immune response to growing cancer in humans that might then lead to an effective cancer immunotherapy. At that time, there was substantial controversy considering the entire field of tumor immunology. There was no effective immunotherapy for cancer patients, and there was considerable doubt as to whether human cancers expressed antigens that could stimulate an immune response. An article in the British Journal of Cancer at the time I moved to NCI was the subject of much discussion because it concluded that no immune response had ever been shown to occur in reaction to a spontaneously arising cancer in animals or man and that results from the study of transplanted tumors in mice had little relevance to human cancers (Hewitt et al. 1976). One prominent quote published in the journal, Cancer Research stated that “it would be as difficult to reject the right ear and leave the left ear intact as it is to immunize against cancer.”

At that time important information regarding the immunogenicity of transplanted tumors in mice had been derived from a variety of investigators including Alexander Fefer, Karl and Ingegerd Helstrom, George Klein, Richard Prehn, Lloyd Old, and many others. These workers had demonstrated that an immune response that prevented the growth of transplanted cancers could be stimulated in mice, but little was known about the therapeutic impact of immune responses on established growing tumors. At that time, it appeared possible that there was no such thing as an immune response to spontaneous cancers in humans and this fear had led few investigators to explore this. Our initial studies were devoted to attempting to measure antibody responses to cancer in patients without success. Although it was suspected that T-cell reactivities would be more important in the rejection of established tissues, it was very difficult to study lymphocytes in vitro because of their very limited survival following removal from animals or humans. Because it was difficult to manipulate T-cells outside the body, I then desperately began another naïve experiment, attempting to see whether or not animals could be immunized against human cancer antigens and give rise to T-cells that could be used for therapy. Even though these xenogeneic cells would have very little survival in vivo perhaps they could temporarily impact on tumor growth. An English scientist named M.O. Symes had published work immunizing pigs with human tumor tissue and utilizing those pig lymphocytes for adoptive cell transfer immunotherapy (Symes et al. 1973). My good friend, David Sachs, a brilliant scientist, then a principal investigator working at the NIH on transplant immunology, had developed a colony of mini pigs partially inbred at the major histocompatibility (MHC) locus. I implanted a human tumor into these pigs, harvested lymphocytes from draining lymph nodes and transferred them to patients. Norman Wolmark, a Fellow in the lab, who later became a prominent surgical oncologist, conducted many of these studies. There were no complications due to the adoptive cell transfer in the six patients we treated but no anti-tumor responses. I strongly suspected this would not work, but I was desperate to do something to help make progress. I hung a sign over the door of my laboratory (paraphrasing a quote from Louis Pasteur) that read “Chance favors the preferred mind, only if the mind is at work.”

In September 1976 when I was working on mouse models and the pig effort, Doris Morgan, Francis Ruscetti, and Robert Gallo published a paper in Science describing a growth factor produced by lymphocytes that could be used to grow healthy human T-lymphocytes in the laboratory (Morgan et al. 1976). This T-cell growth factor later known as interleukin-2 (IL-2) had enormous significance for the work I wanted to do. Shortly after reading this paper, I switched laboratory efforts to determine whether we could grow human lymphocytes in vitro with the goal of directing these reactivities against cancers. Producing sufficient IL-2 for experiments using mouse and human lymphocytes was difficult and it took about 4 months for us to develop approaches to produce IL-2 from mouse spleens and subsequently from human spleens and human peripheral lymphocytes (Rosenberg et al. 1978a, b, 1980a; b; Yron et al. 1980; Strausser and Rosenberg 1978). My lab was small at that point with two post-doctoral fellows, surgeons who had come to the NIH to receive training, as well as two technicians, Susan Schwartz and Paul Speiss, who with me were conducting these experiments. Progressing by tiny increments, we developed robust methods for producing IL-2 from mixed lymphocyte cultures in mice and humans as well as by the stimulation of lymphocytes with lectins to non-specifically induce T-cells to secrete IL-2 in the medium. Gradually our ability to purify IL-2 improved, and we developed procedures to eliminate lectin present in supernatants so that the cultures would not be non-specifically stimulatory (Spiess and Rosenberg 1981). After a long discussion with my wife, Alice, I remember writing down my research plans for utilizing T-cells for immunotherapy and broke it into five sequential studies that if successful could potentially lead us to attempts to utilize T-cells for human immunotherapy. The five steps were as follows:

1. Could we grow T-cells from both animals and humans in culture and enable them to maintain their specific killing activity?
2. Could those reactive T-cells grown in culture be infused into an animal and maintain their killing function?
3. Could we find cells in tumor bearing animals and humans that specifically attack their cancers, grow these cells and maintain their killing activity in culture?
4. Could we infuse those cancer reactive T-cells back into animals where they maintained their killing activity in vivo and retard or eliminate tumor growth?
5. Could we do all of this in humans?

We began these experiments at a very basic level by starting with a simple model using mixed lymphocyte cultures to develop T-cells capable of recognizing allogeneic cells based their expression of MHC antigens. We showed that these alloreactive T-cells generated in vitro would sustain their alloreactivity when grown in culture in both mice and humans (Rosenberg et al. 1978; Lotze et al. 1980a). We then set up a simplified system to test whether these cells were active in vivo by exploring the ability of these transferred alloreactive cells to impact the rejection of skin grafts in mice. Maury Rosenstein, a post-doctoral fellow was able to show that skin graft rejection could be hastened by the transfer of specific alloreactive cells generated and grown in vitro thus demonstrating that these cells when injected into animals retained their specific immunologic reactivity and could reject vascularized tissue (Rosenstein et al. 1981). Our next challenge was to attempt to identify cells from tumor bearing mice as well as people with cancer that could recognize and attack their cancers and grow those cells in vitro to see if they maintained anti-tumor killing activity.

As these technical studies were proceeding, we attempted to grow lymphocytes from the stroma of growing tumors in both mice and humans. Intuitively it appeared that the most likely site to find T-cells reactive against tumor were within the tumor deposit itself. An interesting observation followed. As we put single cell suspensions made from tumors into culture with IL-2, the tumor infiltrating lymphocytes (TIL) grew and slowly cleared the culture of all extraneous cells including tumor cells (Yron et al. 1980; Lotze et al. 1981). After about 2 weeks of culture, pure populations of lymphocytes could be derived. Peripheral blood cells, grown in IL-2 for as short as 3 days, also developed the ability to kill cultured tumor cells in vitro (Grimm et al. 1982). IL-2 was one of many lymphokines, so we named these cells lymphokine-activated killer cells (LAK) and based on this in vitro killing activity, we began studies in mice to see if these LAK cells were capable of killing tumor in vivo (Mule et al. 1984, 1985, 1986; Yang et al. 1985). These experiments using mouse and human LAK cells then consumed us for about 2 years as we demonstrated that tumors induced in the lungs of mice by the intravenous injection of tumor cells could be successfully eliminated by infusion of LAK cells administered up to 3 days after tumor injection. Although it took us longer than it should have, we demonstrated that once tumors became large and vascularized, LAK cells had little to no impact and this eventually turned out to be a false lead in our studies. In the midst of these studies, in 1980, we performed our first studies of LAK cells in humans to study their distribution in vivo (Lotze et al. 1980b). We later administered LAK cells produced by growth in our mammalian IL-2 and labelled with Indium-111 to follow LAK cell traffic in humans with cancer (Fisher et al. 1989; Griffith et al. 1989). The cells trafficked to the lungs and slowly cleared to the liver and spleen over the next 48 h. No anti-tumor responses were seen.

At about this time we began work with an animal tumor model utilizing the transplanted FBL-3 lymphoma that was being studied by Alex Fefer and his group at the University of Washington in Seattle. The FBL-3 cancer had become almost as foreign as a skin graft and thus gave us access to lymphocytes from a mouse that could recognize this tumor. Timothy Eberlein, a surgical fellow in the laboratory took on the project to determine whether the infusion of FBL-3 reactive lymphocytes grown in vitro could successfully treat a mouse with growing FBL-3 lymphoma. Phil Greenberg and other scientists working with Alex Fefer at the University of Washington had elegantly shown that the adoptive transfer of these lymphocytes could mediate the regression of this mouse tumor when both were injected intraperitoneally and thus put in close contact with one another (Cheever et al. 1981; Greenberg et al. 1981). We began studies to see if syngeneic lymphocytes grown in vitro and systemically injected intravenously could also mediate tumor regression. These experiments showed us that vascularized mouse tumors could be made to regress although the antigens being targeted were very likely allogeneic and thus only a poor avatar of true cancer antigens, (Eberlein et al. 1982) if indeed these antigens existed in the human.

## Origins of IL-2 administration as an approved cancer immunotherapy in humans

As we attempted to grow T-lymphocytes with anti-tumor activity, the supplies of IL-2 were an ongoing problem. We were producing IL-2 from stimulated human lymphocytes as well as from a Jurkat tumor line grown in large vats. By July 1982 we had achieved a 1000 fold concentration of naturally produced IL-2 and when injecting this into mice, we saw modest decreases in tumor growth (Rosenberg et al. 1985a). Thus it appeared that IL-2 administered systemically in experimental animals could stimulate immune reactions likely due to the in vivo stimulation of preexisting specific anti-tumor T-cells. Because of these results, we wanted to give IL-2 to patients but getting enough IL-2 was a problem. We could barely make enough purified murine IL-2 to run the most preliminary experiments in mice. Treating patients would require quantities hundreds of times larger.

At this point it became clear we would have to turn to other sources to get large amounts of the IL-2 necessary to investigate its impact on patients. Richard Robb, a scientist at Dupont, purified human IL-2 from a Jurkat leukemia IL-2 cell line that was considerably purer than the concentrated material we had obtained in our own laboratory (Robb et al. 1983). We began discussions with Dupont and by early February 1984, they agreed to supply us with 100 mg of purified IL-2 and the NCI approved a clinical protocol to administer IL-2 as an investigative new drug in clinical experiments. In June 1983, Richard Robb, from Dupont, came to Bethesda and handed us six vials containing 75 mg of purified IL-2. Mike Lotze who was leading many of these early human experiments then tested this IL-2 in vitro as well as in mice to determine its safety (Lotze et al. 1978; Donohue et al. 1984). Interestingly, as a requirement for providing us IL-2, Dupont asked us to guarantee that we would continue using their IL-2 as long as it was “suitable” even if other sources of IL-2 became available using recombinant DNA technology. We refused to agree to that stipulation and Dupont eventually conceded and agreed to supply us with purified IL-2. In July 1983, we began the first phase 1 trial using natural IL-2. Due to limited material, we could not dramatically escalate the dose although we did treat 16 patients with advanced cancer and no anti-tumor activity was seen in any patient.

Thus by early 1984 we had treated 26 patients with metastatic cancer, and all had developed progressive cancer and died of their disease. This included the one patient that received a transfusion from another cured patient, six patients that received pig lymphocytes sensitized to human cancer tissues, three patients that had received LAK cells generated in mammalian IL-2 and sixteen patients that were treated with the Dupont natural mammalian IL-2, none of whom had shown any sign of clinical response. But as these treatments were progressing, the advent of recombinant IL-2 was about to make more IL-2 available for mouse and human experiments.

My work with IL-2 was based on our hypothesis that simulating T lymphocytes from cancer patients could potentially activate endogenous lymphocytes with anti-tumor activity that could destroy growing tumor cells. This was both a frustrating and exciting time in this effort. Thus far we had seen no tumor shrinkage to IL-2 administration and until we saw a response, it was not at all clear that our hypothesis was correct and that it would ever happen. The amounts of IL-2 available had been limiting and multiple biotechnology companies were in a race to clone the gene encoding IL-2, including Hoffman-La Roche, Amgen, Genentech, Dupont, and a small startup biotechnology company called Cetus Immune. A Japanese scientist, Tadatsugu Taniguchi, was the first to clone the gene for IL-2 isolated from a Jurkat cell line in 1982. He inserted this gene into monkey cells that began making prodigious levels of IL-2. He announced this work at scientific meetings and shortly thereafter published the gene sequence of IL-2 in Nature (Taniguchi et al. 1983). I had never heard of Cetus when I received a call from Gary Fathman who asked me to come to Cetus to give a lecture on my work. On July 30, 1982, as I was performing experiments with partially purified mammalian IL-2, I lectured at Cetus hoping to stimulate them to produce recombinant IL-2 that I could use in experiments as well as for human treatment. I revisited them again on October 20, 1982 to discuss the mouse experiments we were performing with IL-2 that were showing a modest but direct impact on tumor growth in mice. As their work proceeded, I worked with David Mark, a molecular biologist, and Kirston Koths, a protein chemist, who were the main scientists at Cetus attempting to produce recombinant IL-2. David Mark cloned the gene for IL-2 based on its known sequence and inserted it into bacteria that began producing large amounts IL-2 that comprised up to ten percent of all of the proteins produced by these transformed bacteria (Wang et al. 1984) (Rosenberg et al. 1984). In mid-June, when Cetus was holding their annual retreat to summarize the results of the company’s work, I gave a presentation aimed at obtaining small amounts of recombinant IL-2 to begin work in the laboratory. Unbeknownst to me at the time, there was debate at Cetus concerning whether to provide me with small, precious amounts of their recombinant IL-2. In a prior meeting at Cetus following my presentation, an announcement was made to let everyone in the room know that everything that they were about to present was confidential. I had never agreed to this kind of secrecy in medicine, which appeared unseemly when one was trying to develop treatments for desperate cancer patients, and as they requested, I sat in a side room unable to hear their discussion. As I was leaving Cetus, Kirston Koths walked with me to a car to take me to the airport. Apparently, Jeff Price, the president of Cetus, had decided to provide me with some material and before I stepped into the car. Kirston reached into the pocket of his shirt, pulled out a screw top vial containing less than a milliliter of clear liquid and handed it to me. This was recombinant IL-2 that was just coming off purification columns. We transferred the vial of IL-2 from his pocket to a pocket in my jacket, and I returned to the NIH and immediately began experiments. Our first experiments showed that this recombinant IL-2 could indeed grow T cells and generate LAK cells from mouse and human lymphocytes. There was more IL-2 left on the tip of a pipette that we used to deliver this material to cultures than we had previously had available from natural mammalian IL-2. Dupont IL-2 was used in vitro at dilutions of up to 1 to 4, but the recombinant IL-2 could grow cells at dilutions of 1 to 400,000. Laboratory experiments in mice appeared promising, and I was eager to begin testing IL-2 in humans.

Michael Lotze, a smart and hardworking surgical resident, had worked in my laboratory years earlier in the midst of his surgical residency and had returned to our senior staff in the Surgery Branch. He worked closely with me to lead our trials using highly purified natural IL-2 and in March 1984, we were performing the last of the clinical treatment of patients with natural IL-2 from Dupont. While these trials were ongoing, I had begun seeking FDA approval to use the recombinant material, that we were receiving in increasing doses, from Cetus in clinical trials based on its effectiveness in animal models. We received approval from the FDA on January 17, 1984, just 7 months after I was handed the first vial of recombinant IL-2 and we began human experiments. We demonstrated that the half-life of IL-2 in people was only five to seven minutes, which indicated we might need very large amounts of IL-2 that only recombinant material could make available (Donohue and Rosenberg 1983). Our trials went in two directions. We gave either recombinant IL-2 alone or LAK cells that had been produced with recombinant IL-2. The first patients in our phase one trials with recombinant IL-2 began in March of 1984. Dr. Vincent Devita, then the Director of the National Cancer Institute, provided us with the space and resources needed at the NCI to pursue this work. We treated 23 patients with advanced cancer using recombinant IL-2 in increasing doses, and 27 patients with LAK cells produced with recombinant IL-2, alone or along with IL-2. No patients exhibited tumor shrinkage and all subsequently died of their cancer. In these trials of IL-2 administration we began to see the toxicities that came to characterize what later became known as the “cytokine release syndrome”. Patients developed high fevers to 104 degrees Fahrenheit and chills that could be abrogated by the administration of anti-inflammatory agents such as indomethacin and acetaminophen. Transient hypotension and renal dysfunction became prominent. We continued to escalate the doses of IL-2 based on pharmacokinetic studies of its short in vivo half-life without clinical effect. Thus after treating 76 patients without success, and feeling somewhat desperate to fully evaluate the possibility that this approach could stimulate immune reactions against cancer, I decided to treat the next patient with the maximum amount of recombinant IL-2 the body could tolerate by giving IL-2 every 8 h so that blood levels would be continuously sustained at levels sufficient to stimulate the trimeric IL-2 receptor on lymphocytes.

**Fig. 1:**
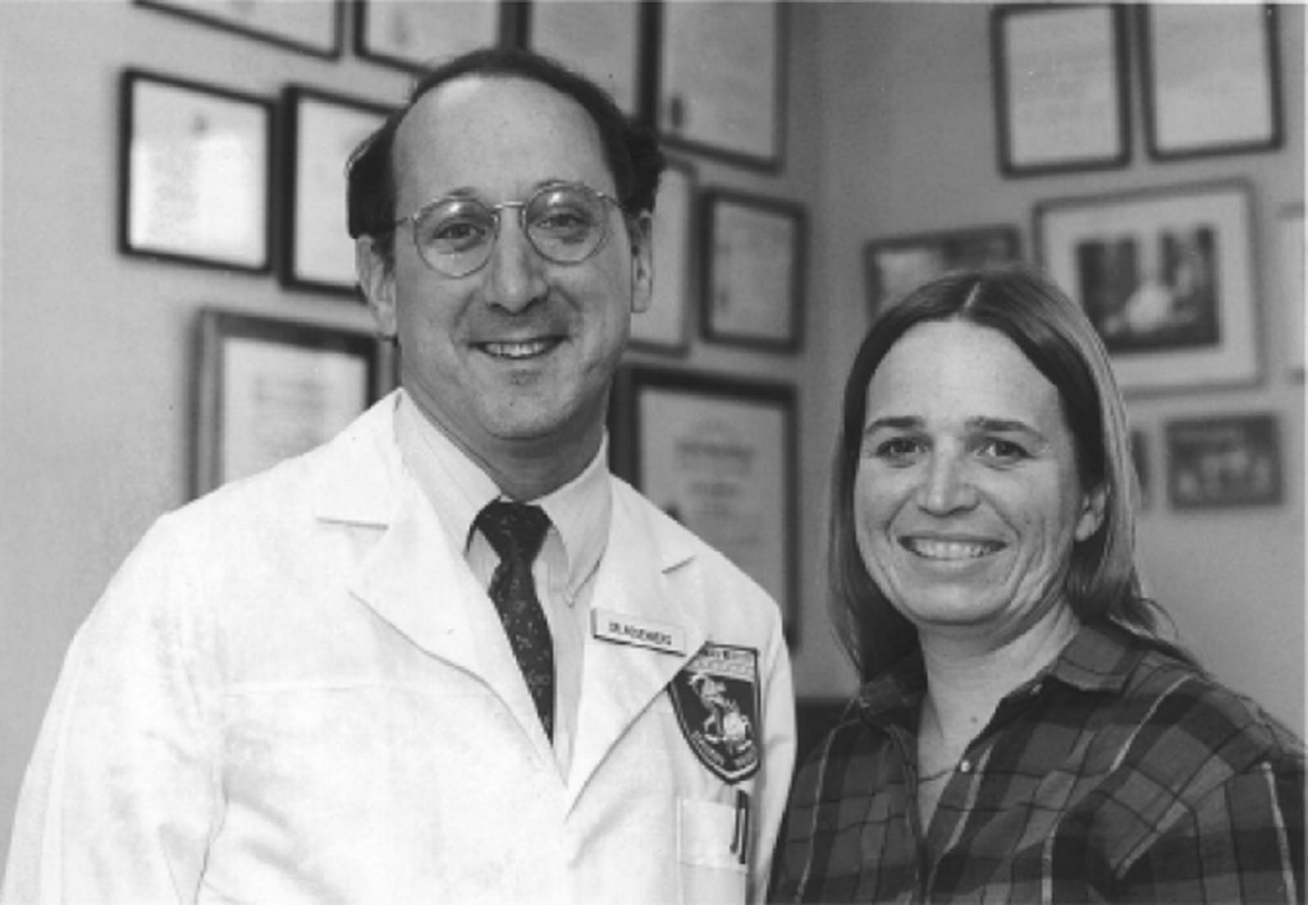
With the first patient to respond to IL-2 administration. Her metastatic melanoma underwent a complete regression in 1984 and she remains disease-free over 36 years later

**Fig. 2:**
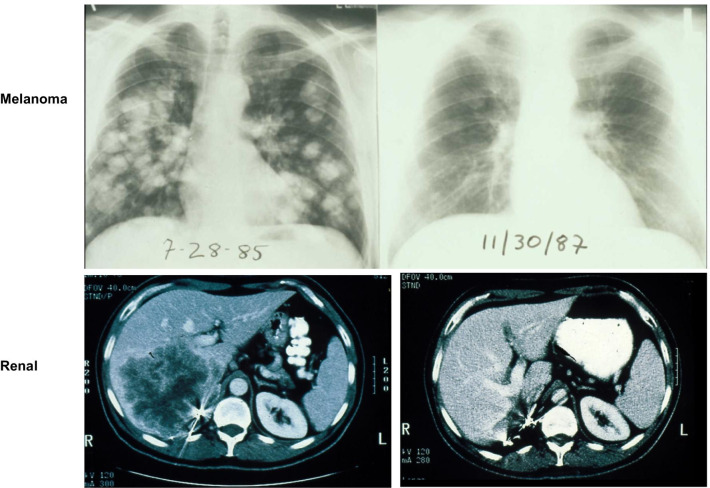
Examples of the earliest complete responses to IL-2 administration that ultimately led to the first FDA approval of a cancer immunotherapy (upper panels, melanoma; lower panels, renal cancer)

Utilizing this highly aggressive treatment with IL-2, the first patient we treated was a 33-year-old Navy officer with extensive subcutaneous metastatic deposits from a melanoma (Rosenberg et al. 1985b). She first came to an NCI funded group in Frederick, Maryland and had received several experimental therapies including monoclonal antibodies and alpha interferon with no effect. We treated Linda aggressively with LAK cells along with high-dose intravenous IL-2 injections every 8 h starting on November 29, 1984. At this aggressive schedule with IL-2, significant toxicities were seen including capillary leak syndrome which primarily affected the lungs and led to symptoms similar to those of pulmonary edema. We treated Linda with IL-2 to maximal tolerance and she gained 22 lb of fluid in the first 2 weeks after we began treatment due to fluid leaking into the soft tissues of her body. Her symptoms were severe enough that she had to be intubated overnight to receive enough oxygen and with maximum medical support, she slowly recovered. By the time Linda was discharged, we saw no change in the size of any of her subcutaneous tumors. This remained the case on January 9, 1985 when she returned for her first follow up appointment. We biopsied one lesion and several days after she returned home from this first visit, we received the pathologist’s report on the biopsy that noted the appearance “of tumor cell ghosts is consistent with patients previously diagnosed malignant melanoma…no viable tumor is identified.” We called the patient to tell her of this result, though she told us that none of the many tumors that she had over her body had shown any change. When she returned for her second follow up visit a month later, her tumors were dramatically shrinking, and she went on to have a complete disappearance of all of her metastatic melanoma (Fig. 1). “Eureka” moments are rare in research, but this was one for me. For the first time, an immunologic maneuver had caused the regression of cancer in a human. Linda remains disease free now over 35 years later. We treated 25 patients with recombinant IL-2 and LAK cells, and we published our findings in the New England Journal of Medicine in 1985 (Rosenberg et al. 1985b). Linda and the other patients that responded in this trial were the first patients to develop reproducible tumor shrinkages from any immunotherapy. These were heady times and in Fig. 2 are some examples of these early responses. The New England Journal article attracted considerable attention and many others began working on this method of stimulating the body’s immune system.

Although I knew our findings with IL-2 would attract attention, I was surprised and embarrassed by the outbreak of world-wide reporting about this approach. Enthusiasm concerning this work was exacerbated by an article in Fortune magazine that appeared 10 days before the New England Journal was scheduled to publish our article. The magazine ran a cover story, the headline of which was “Cancer Breakthrough”. I had been contacted by the author of the article and refused to speak with him until the New England Journal of Medicine article was published as was reported in the Fortune magazine article. I assume results had been leaked by executives at Cetus though this was never determined. In addition this added publicity occurred because I had gained some recognition earlier in that year as one of the operating surgeons that removed President Ronald Reagan’s colon cancer at Bethesda Naval Hospital. The publicity led to an influx of patients, and we began treating up to four patients a week. IL-2 administration mediated cancer regression in patients with melanoma and renal cell cancer who were included in the first 25 patients reported in the New England Journal. But the toxicity was severe. In ongoing studies, the 35th patient to receive LAK cells plus IL-2, a 24-year-old boy with metastatic melanoma, was the first patient to die from this treatment. As we treated additional patients, we began to learn how to manage the toxicities and to learn the limits of IL-2 administration. There was considerable criticism of our approach because of the toxicity caused by the treatment. Although our IL-2 treatment related mortality in patients with advanced metastatic cancer refractory to all other treatments was 3–4% in our first 200 patients, we ultimately decreased this toxicity level to 0.5% (Kammula et al. 1998). Linda Taylor and the many subsequent patients had received high-dose bolus IL-2 along with LAK cells but the relative contributions of these two treatments were unclear.

As is often the case when reports of new approaches to cancer treatment appear, there was considerable controversy concerning our early results. Dr. Charles Moertel, a prominent oncologist at the Mayo Clinic, wrote an editorial in the Journal of the American Medical Association in December 1986 where he argued that initial studies in a small number of patients often claimed great success even though these treatments “were subsequently discarded as ineffective.” He further argued that the toxicities and expense “are not balanced by any persuasive evidence of true net therapeutic gain. This specific treatment approach would not seem to merit further application in the compassionate management of patients with cancer.” But others recognized the importance of this first effective immunotherapy. John Durant, President of the Fox Chase Cancer Center in Philadelphia, wrote an editorial for the New England Journal of Medicine that reported “…perhaps we are at the end of the beginning of the search for successful immunotherapy for cancer” paraphrasing Winston Churchill’s war time remarks at the victorious conclusion of the North African campaign during the second World War.

After treating patients with LAK cells and IL-2 that mediated objective cancer regressions in 15–20% of patients with metastatic melanoma and renal cancer (about one-third were complete regressions), we performed a prospective randomized trial in which 97 patients with metastatic renal cell cancer and 54 patients with melanoma, were treated with IL-2 with or without LAK cells (Rosenberg et al. 1993). We saw no differences in response rate or survival in patients in these two groups and we then eliminated LAK cells from our treatment. It appeared clear that IL-2 administration had mediated the cancer regressions we were observing and that the therapeutic impact of IL-2 was due to its ability to stimulate lymphocytes that expressed IL-2 receptors. We went on to treat over 1000 patients with metastatic melanoma or renal cancer using high-dose IL-2 and achieved objective responses in approximately 15% of patients, 1/3 of which were durable complete regressions and very few of these latter patients have recurred with follow up over 25 years later (Smith et al. 2008; Rosenberg et al. 1994a). There was much that we then tried that did not improve our treatment. We performed a randomized trial of patients receiving either high-dose IL-2 alone or IL-2 plus combination chemotherapy in 102 patients with metastatic melanoma and saw no difference in survival rates (Rosenberg et al. 1999). Dr. James Yang of our Surgery Branch Senior Staff, who was a close partner in much of this work, performed a three-armed randomized trial in 156 patients with metastatic renal cancer to evaluate either high-dose Il-2, a lower dose of IL-2 or a low dose of daily subcutaneous IL-2 (Yang et al. 2003). There was a strong suggestion from these trials that high-dose IL-2 had a superior response rate, and we thus settled on this high-dose regimen in subsequent trials. Dr. Vincent DeVita, then Director of the National Cancer Institute, recognized the importance of this work and supported a consortium of outside investigators to facilitate these clinical studies that led to the treatment of 270 consecutive patients with metastatic melanoma treated between 1985 and 1993 using high-dose bolus IL-2 that provided response rates and survival similar to those we originally reported (Rosenberg et al. 1994a; Atkins et al. 1999). In this trial 6% of patients with metastatic melanoma achieved a complete regression of all disease and 10% of patients experienced a partial cancer regression. These results and our prior trials of the effectiveness of high-dose IL-2 administration provided convincing evidence and ultimately led to the Food and Drug Administration approval of this treatment as the first immunotherapy approved for patients with cancer. The FDA approved high-dose IL-2 for the treatment of patients with metastatic renal cancer in 1992 and for melanoma patients in 1998.

## Origins of cell transfer immunotherapy for human cancer

As we were seeing the first responses to IL-2 in Linda Taylor, we began experiments to understand the cellular mechanisms underlying the phenomenon. Intuitively, it seemed that lymphocytes with putative specific anti-tumor activity (in contrast to the non-specific activity of LAK cells) would most likely be found within the stroma of a tumor deposit. Critical to the establishment of any cell transfer immunotherapy was the identification of immune cells capable of specifically reacting with the cancer.

We began experiments to see if we could detect specific tumor reactivity from tumor infiltrating lymphocytes (TIL) present in freshly resected tumors and grown for prolonged periods in IL-2. Paul Spiess, a technician who was working in my laboratory began studies in mouse methylcholanthrene-induced sarcomas that were being serially transplanted in the laboratory. Cell suspensions from growing murine sarcomas were cultured in IL-2 for several weeks at which time they had cleared tumor cells from the culture. We began experiments treating mice with these growing TIL cells compared to LAK cells that were produced over the course of a few days. LAK cells had virtually no impact on large and vascularized established nodules in the lung. However, TIL grown for extended periods in vitro in IL-2 sometimes specifically killed only the tumors from which they were derived and would not kill other tumors thus suggesting that the specific killing by TIL was targeting unique cancer related antigens that distinguished the tumors from normal tissue. In July 1986, about 18 months after our first studies of TIL, we published our first paper on this phenomenon in the journal Science (Rosenberg et al. 1986). The last sentence of this paper stated: “Experiments with murine tumors such as those described in this report can provide the rational for combining these therapeutic approaches to develop optimal combination immunotherapies for the treatment of cancer in humans.”

As these mouse experiments were being performed, Suzanne Topalian and then Linda Muul, a post-doctoral fellow in the laboratory performed experiments in which TIL cells grown from a human melanoma recognized only the tumor from which they were derived and not tumors from other patients (Topalian et al. 1989; Muul et al. 1987). These experiments appeared to demonstrate that human cancer antigens did indeed exist. It was a second Eureka moment that led us to pursue the impact of TIL cells in humans even though we had not identified their molecular nature. We quickly began a pilot study of the impact of autologous TIL administered to patients with metastatic melanoma. Suzanne Topalian, a surgeon and new fellow in the laboratory, developed techniques to grow these human TIL in large numbers, and we received permission from our investigational review board and the FDA to utilize relatively small numbers of these cells for the treatment of 12 patients with metastatic cancer. No clinical anti-tumor responses were seen in the treated patients (Topalian et al. 1988). We then accelerated these studies and established a separate laboratory strictly devoted to growing TIL in large numbers. In a mammoth effort, we began growing TIL in tissue culture plates and then in over 70 culture bags to grow up to 200 billion lymphocytes that we could use for patient infusion in conjunction with IL-2 to support the cells in vivo. Nine of 16 patients with metastatic melanoma, who had not been treated with prior IL-2 alone, showed partial or complete cancer regressions about twice the response seen to IL-2 alone. Two of five patients refractory to prior IL-2 treatment also responded. We published the results of the treatment of these first 20 patients with this combined cell therapy in the New England Journal of Medicine in December, 1988 (Rosenberg et al. 1988). This work demonstrated that the action of T-lymphocytes was at least in part responsible for mediating tumor regression in humans with advanced cancer.

## Origins of gene therapy for cancer

From the beginning of our work with T-cell transfer, I considered whether it would be possible to improve the activity of TIL by genetically modifying them to express properties essential for tumor identification and destruction of cancer cells. Our first experiments to genetically engineer TIL began after I approached an NIH scientist named Werner Green at the NIH who had identified the gene that encoded IL-2 receptors on T cells. These experiments were based on the hypothesis that by genetically creating T cells expressing a large number of IL-2 receptors we might make these cells more responsive to IL-2 administration and therefore more effective in destroying tumors. Although it seemed a reasonable idea at the time, we did not succeed in getting the gene for the IL-2 receptor into TIL using the calcium phosphate transfection methods that were then in use.

**Fig. 3:**
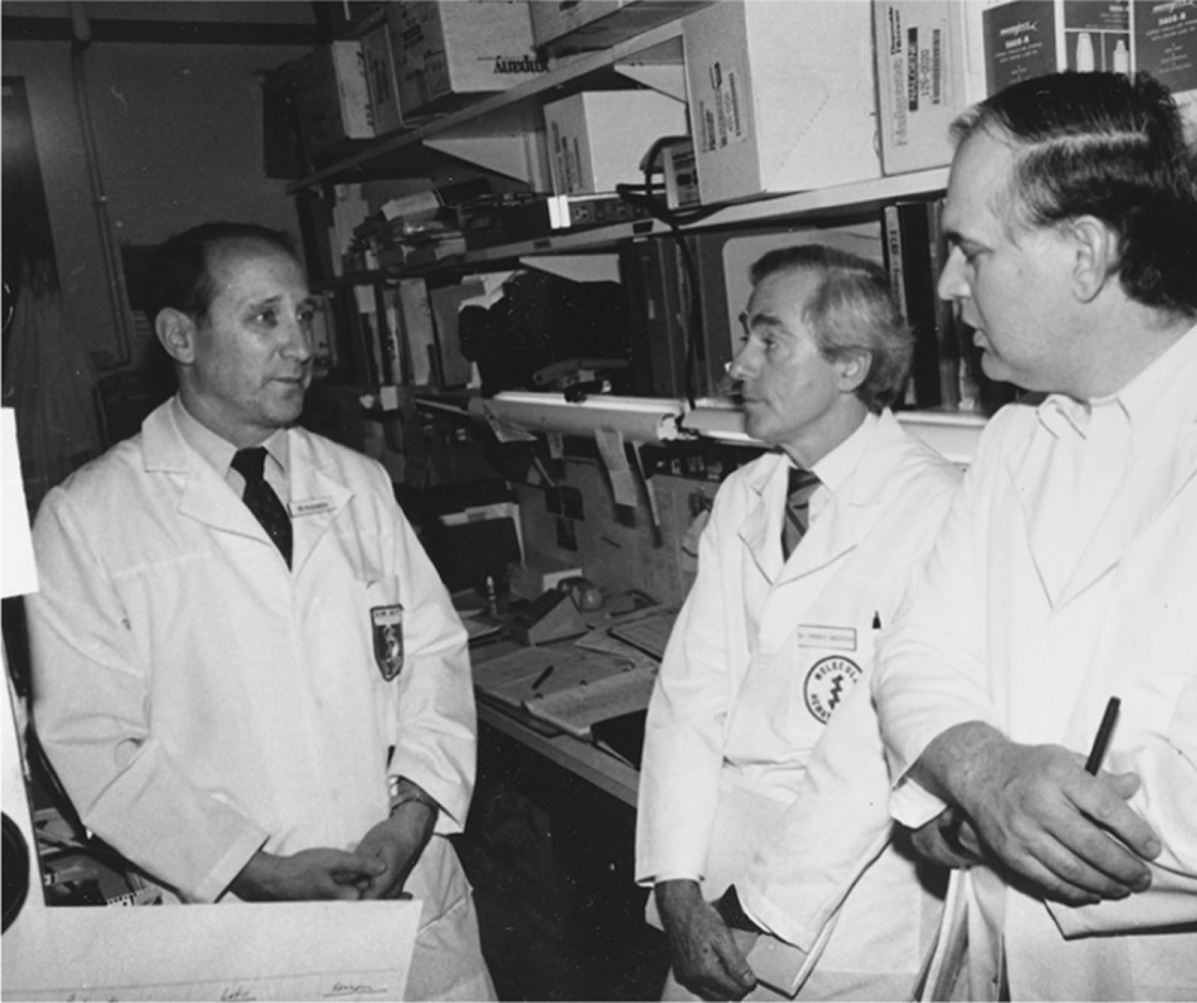
One of many discussions I had with W. French Andersen and Michael Blaese in 1989 as we planned and conducted the first introduction of a foreign gene into humans

Although our earliest attempts at gene modification of lymphocytes were unsuccessful, I began a collaboration with two prominent NIH scientists, Drs. French Anderson and Michael Blaese, who were working to correct gene deficiencies in children with adenosine deaminase (or ADA) mutations that prevented lymphocytes from functioning normally. French and Mike had been working with retroviral approaches as a more efficient way to introduce genes into cells, and it appeared that this might be an important technical advance that would enable us to genetically modify lymphocytes for administration to patients. Although I was mainly interested in inserting therapeutic genes into TIL, no one had ever introduced any foreign genes into humans. After multiple discussions and many dozens of laboratory meetings to discuss new data, we decided that one way to “break the ice” to enable gene introduction into humans, would be to use a marker gene that would enable us to track TIL once they were injected in vivo (Fig. 3). We therefore proposed a clinical protocol in which we inserted a bacterial gene encoding the enzyme neomycin phosphotransferase that would enable us to distinguish the administered cells from normal endogenous lymphocytes in that patient. In close collaboration with French Anderson, Mike Blaese, and many others, we slowly began to show success in inserting this bacterial gene into human cells with high efficiency and growing them to large numbers.

There was considerable regulatory and societal concern about inserting new genes into human cells and passionate arguments were raised by many that this was too great a risk to patients, healthcare scientists and the public. Objections included the fear that the retroviruses, engineered so that they could not replicate but could insert transgenes might recombine with endogenous viruses and gain properties that enabled them to replicate, infect other cells, and perhaps cause cancer. Some lay members of the review groups raised spiritual concerns that tampering with DNA, the essence of “god’s creation” was immoral.

The review process to gain permission to perform this first gene introduction into humans was an exhausting and a painfully slow experience as we presented data to each review group to overcome these objections. We were unable to retrovirally insert genes into mouse lymphocytes despite major efforts and this limited the kinds of testing that we could perform in mice in vivo. We developed a clinical protocol and in June 1988, we presented our proposal to the Investigational Review Board panels from several Institutes at the NIH to seek approval for this human experiment. We next presented our plans to the NIH Biosafety Committee that dealt with recombinant DNA use and in July, we presented to the Gene Therapy Subcommittee of the Recombinant DNA Advisory Committee (RAC) that had been established years earlier to ensure the safety of any work with recombinant DNA. Our presentations were met by major objections by each of the review groups and each time we made changes to respond to these objections, repeat review by the prior groups was required. We emphasized the safety of our procedures and all of the modifications we had made to prevent potential mishaps. We emphasized that the genes were being inserted into somatic cells and could not enter the germ line and affect future generations. We wanted to treat patients with advanced cancer who had no other treatment alternatives and had life expectancies of 3 months or less. This clinical effort might teach us much that could be applied to develop and improve cancer treatments. There was heated discussion at the RAC before a vote was taken and the protocol was finally approved by a vote of 16 to 5.

The RAC is an advisory committee to the NIH Director, then Dr. James Wyngaardan, and several weeks later on October 18, 1988, Dr. Wyngaardan ruled that a split vote on this first human gene therapy attempt was problematic, and he wanted unanimity in this RAC decision. I thought this was a strange decision because in my dealings with research and clinical efforts even in my own group, I almost never could get unanimous agreement about contentious issues. We then provided more data to the RAC and on December 9 the RAC approved the protocol with a vote of 13-0 with one abstention. A month later in January 1989, we were ready to proceed, but at the next scheduled meeting of the RAC, Jeremy Rifkin, a biotechnology activist, announced he was filing suit to halt the experiment because of an inadequate review process. The meeting was chaotic. Many handicapped people filled the room to support Rifkin’s objections. His argument was an emotional one, and he stated “it is the first experiment in the world in which a foreign gene is to be placed into a human being. With this experiment, we begin the whole era of human genetic engineering…if we are not careful we will find ourselves in a world where the disabled, minorities and workers will be genetically engineered…we will be back next time and the next time.” This delayed our clinical trial by several more months until the NIH settled Rifkin’s lawsuit and thus enabled us to proceed. On May 22, 1989 we treated the first patient by injecting his own TIL cells grown from his tumor and retrovirally transduced with the gene encoding the bacterial enzyme neomycin phosphotransferase. We went on to treat 10 patients with advanced cancer using their gene-modified autologous cells and although none showed a clinical response, we learned a great deal about the traffic and persistence of these gene modified cells that could be recovered from tumor 2 months after cell infusion. This trial demonstrated that administration of genetically modified human cells could be done safely in humans despite the dire predictions of many who objected to this approach. A year later, in December 1990, we published these results in the New England Journal of Medicine (Rosenberg et al. 1990).

## The next 30 years (1990 to 2020)

By 1990, we had established that a strictly immunologic maneuver such as IL-2 administration could mediate the rejection of growing metastatic cancer in humans. Effective immunotherapy for patients with cancer was indeed possible! It was no longer only an intuition or a dream. Further, we had demonstrated that autologous tumor infiltrating lymphocytes (TIL) isolated and grown from the stroma of growing melanoma deposits exhibited specific tumor recognition in vitro and the infusion of these cells could mediate cancer regression. This strongly suggested that these TIL stimulated in vivo by IL2 administration accounted for the anti-tumor impact of IL-2. Finally, we had demonstrated that it was feasible and safe to introduce foreign genes into patients using retroviral transduction of lymphocytes. Although this work involved the introduction of a bacterial gene that would enable us to track the in vivo fate of administered lymphocytes, it opened the possibility that introduction of other genes might improve the anti-cancer properties of administered cells.

**Fig. 4:**
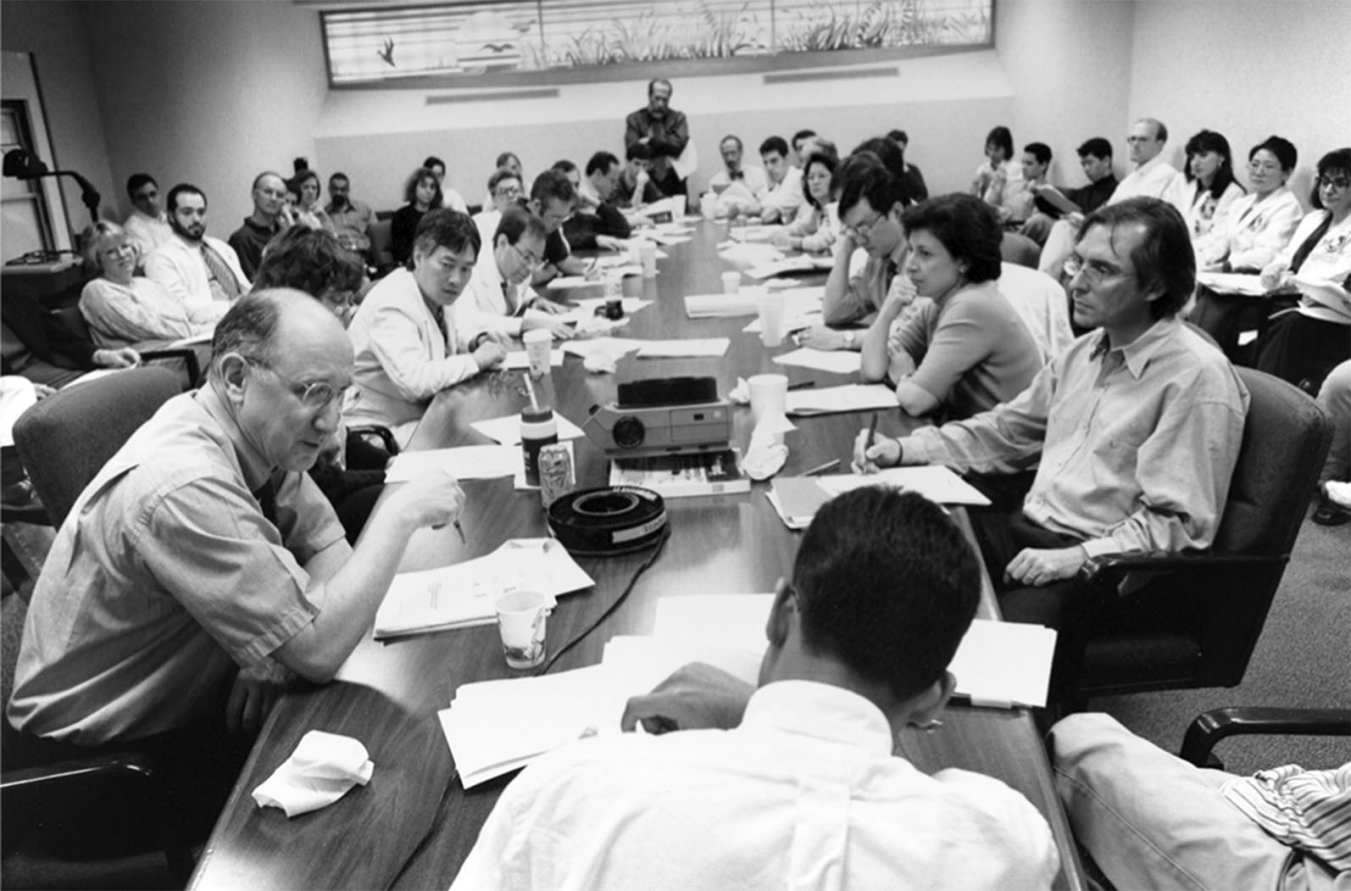
In weekly laboratory meetings (this one in 1998) of all members of my group including scientists, clinical fellows, technicians, students and nurses. Fellows presented their ongoing research designed to receive input and keep all up-to-date on clinical and laboratory efforts

**Fig. 5:**
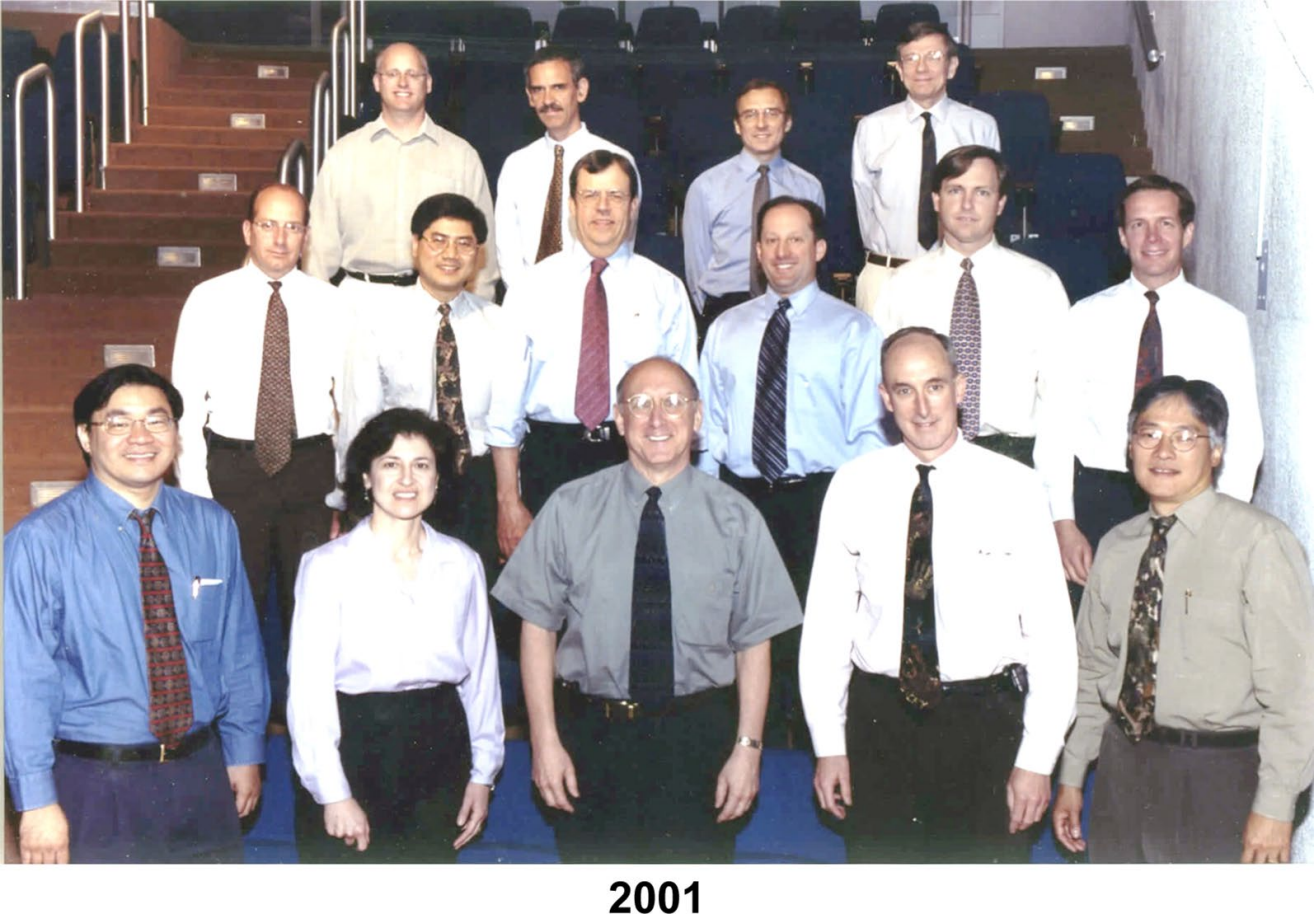
Senior members of the Surgery Branch in 2001. Front row: Patrick Hwu, Suzanne L Topalian, Steven A. Rosenberg, H. Richard Alexander, James C. Yang; Second row: David S. Schrump, Dao Nguyen, David N. Danforth, Steven K. Libutti, David L. Bartlett. Douglas J. Schwartzentruber; Third row: Richard A. Morgan, Paul F. Robbins, Nicholas P. Restifo, John R. Wunderlich

**Fig. 6:**
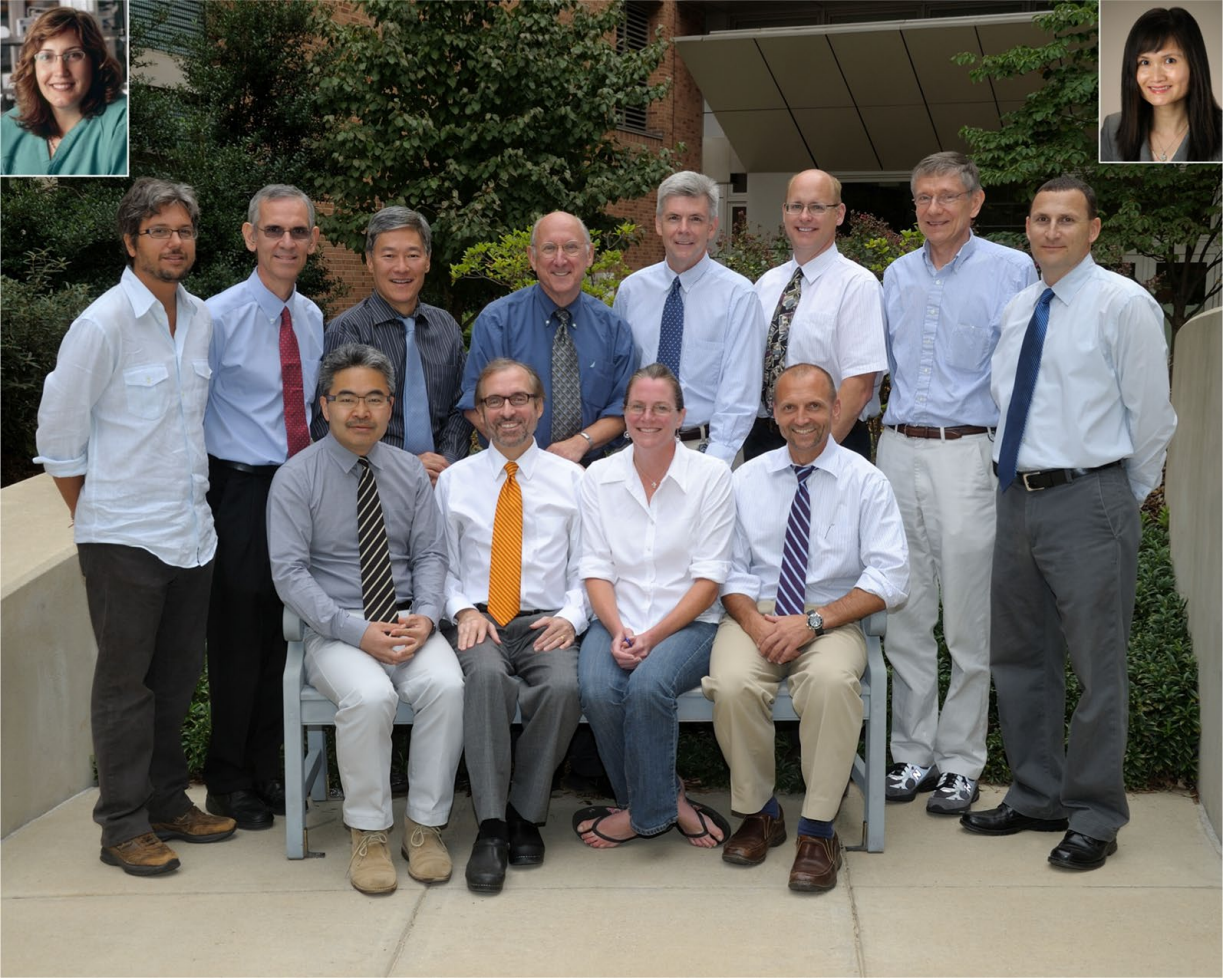
Immunotherapy staff in the Surgery Branch in 2006. Front row: Ken-ichi Hanada, Nicholas P. Restifo, Maria R. Parkhurst, Mark E. Dudley. Second row: Luca Gattinoni, Paul F. Robbins, James C. Yang, Steven A. Rosenberg, Richard M. Sherry, Richard A. Morgan, John R. Wunderlich, Steve A. Feldman. Inserts: Marybeth S. Hughes, Giao Q. Phan

**Fig. 7:**
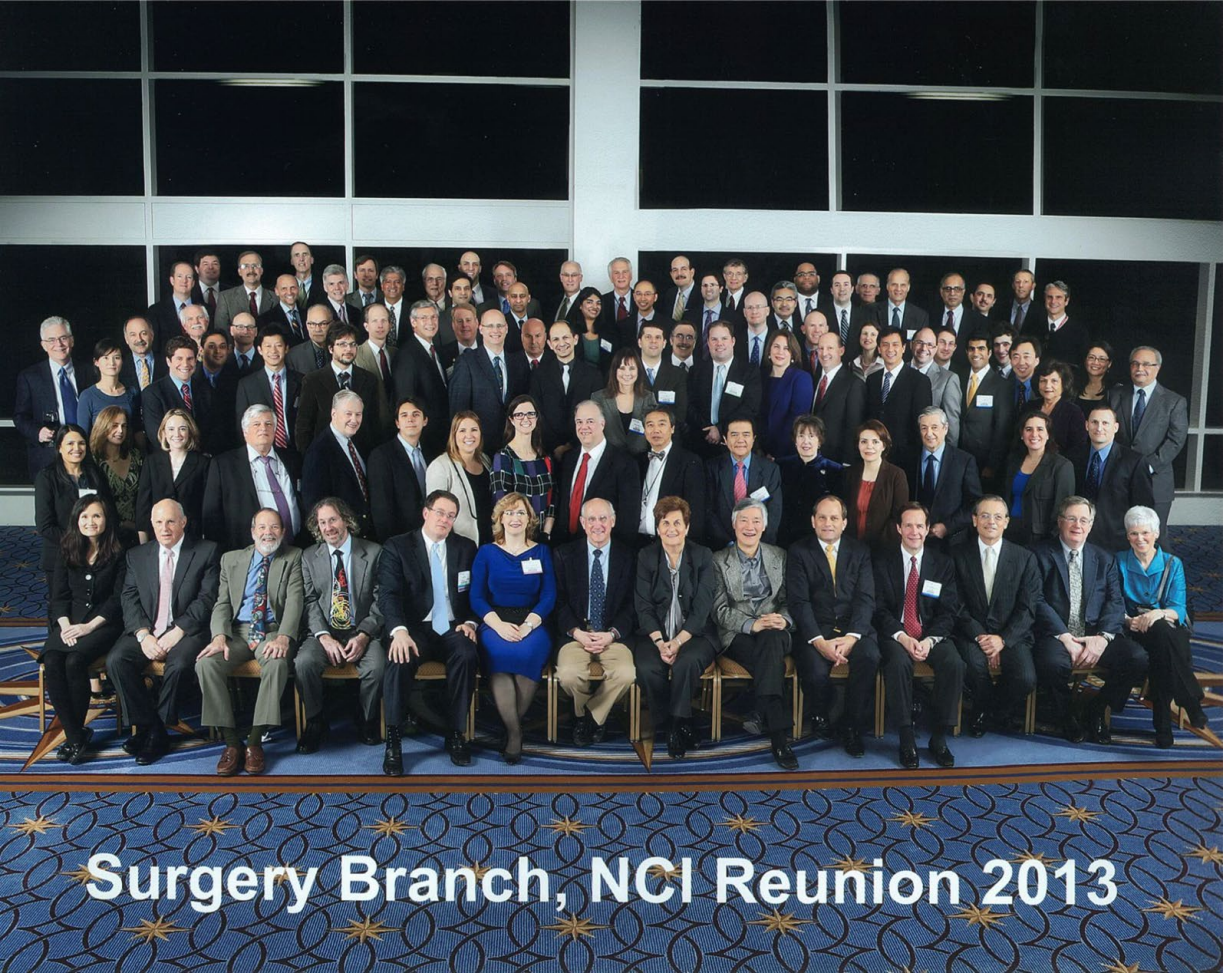
One of many reunions of former fellows and staff of the Surgery Branch we had periodically starting in 1997 (shown here in 2013)

**Fig. 8:**
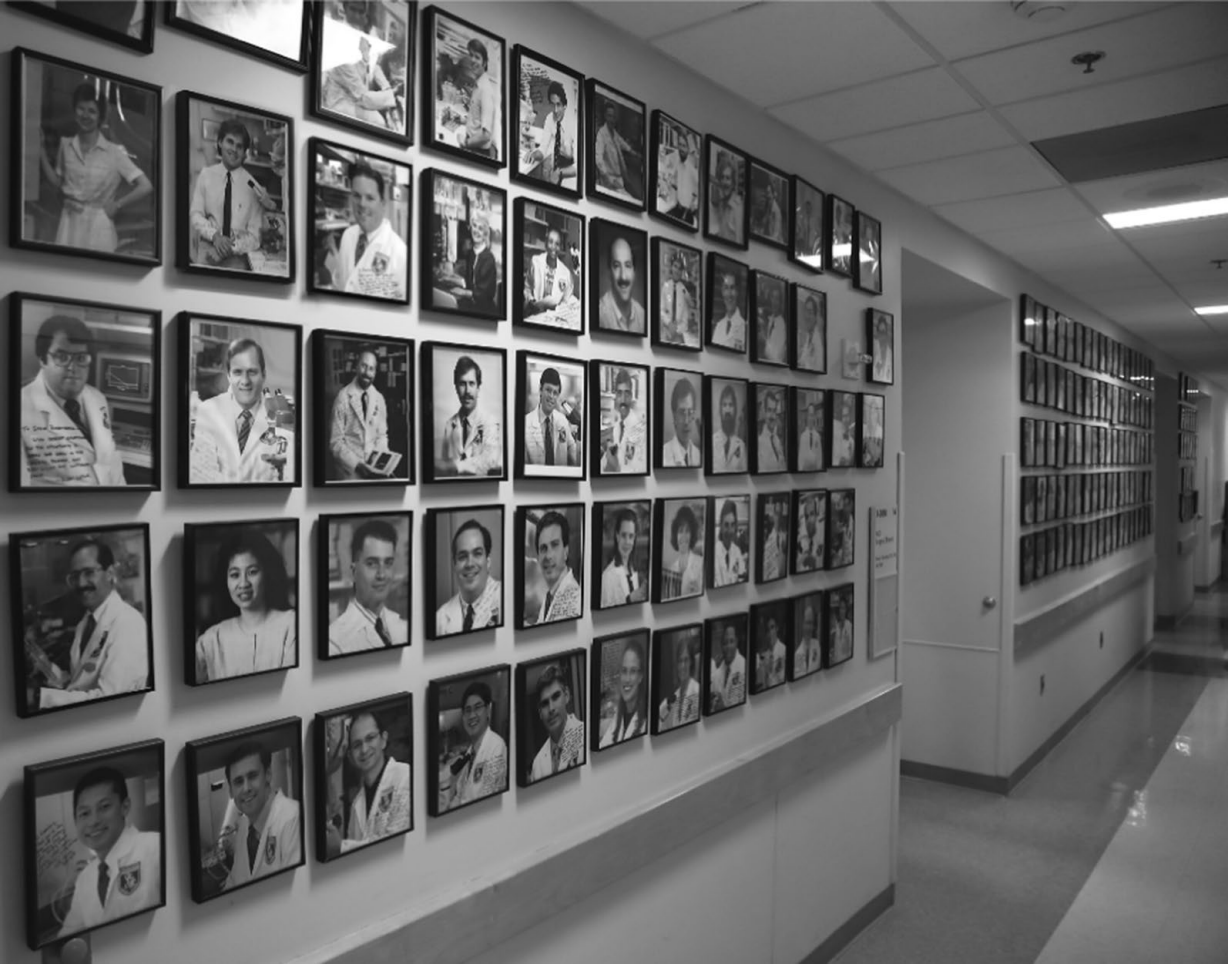
Photos line the walls outside my office dedicated to the hundreds of fellows who worked in my laboratory and make important contributions to our efforts

These early findings were the basis of my work over the next 30 years devoted to improving and expanding the use of cellular therapies to treat patients with metastatic cancer, using either naturally occurring anti-tumor T cells or using genetically engineered lymphocytes (Figs. 4, 5, 6, 7, 8, and Table 1). The following is a brief description of our efforts and progress in these two lines of research.

**Table 1:** Scientists who worked in the Rosenberg laboratory for over 10 years

Baker, Alan	1974–1995
Schwarz, Sue	1974–2013
Spiess, Paul	1976–2005
Lotze, Michael	1978–1980; 1982–1990
Mule, James	1983–1993
Yang, James	1984–present
Topalian, Suzanne	1985–2006
Schwartzentruber, Douglas	1987–2003
Sherry, Richard	1988–1991;1997–2021
Gaye, Michelle*	1988–present
Hwu, Patrick	1989–2003
Restifo, Nicholas	1989–2019
Robbins, Paul	1992-present
Wunderlich, John	1992–2020
Parkhurst, Maria	1994–present
Dudley, Mark	1996–2013
Kammula, Udai	1996–1999; 2004–2017
Morgan, Richard	2001–2013
Hughes, Marybeth	2002–2014
Goff, Stephanie	2005–2009; 2014–present
Feldman, Steve	2006–2018
Somerville, Robert	2009–2019
Lu, Yong-Chen	2009–2021

^*^Administrative

## Use of naturally occurring anti-tumor lymphocytes for the adoptive cell therapy of cancer in patients

Our studies published in 1988 showing that administration of autologous TIL grown from resected tumor nodules of patients with metastatic melanoma represented the clearest example that cell transfer therapy could mediate the regression of metastatic cancer in humans (Rosenberg et al. 1988). Following that report in 20 patients, we went on to treat 86 patients with metastatic melanoma utilizing tumor infiltrating lymphocytes plus IL-2 (Rosenberg et al. 1994b). The overall objective response rate in these patients was 34% and there was no significant difference in objective response rates in patients treated with and without low dose cyclophosphamide or had previously progressed through IL-2. Of the 29 patients that had exhibited an objective clinical response, only 5 were complete and only 2 were ongoing at 21 and 46 months at the time of publication. The median duration of objective partial responders was only 4 months. Further, persistence of the transferred cells in the circulation was short following infusion. Studies using genetically marked TIL revealed that at 1 week barely 0.1% of the lymphocytes in the circulation represented the transferred cells. Indium-111 labeled TIL showed “trapping” of the transferred lymphocytes in the lungs for up to 48 h as the cells then slowly cleared from the lungs mainly to the liver and the spleen (Fisher et al. 1989; Griffith et al. 1989).

Emerging information from ACT studies in murine tumor models revealed a need for a lymphodepleting regimen prior to the cell transfer to mediate improved anti-tumor effects. We next treated patients with a non-myeloablative high dose regimen of cyclophosphamide (60 mgkg × 2 and fludarabine (25 mg/m^2^ × 5) that resulted in a reduction of circulating lymphocytes to near zero. Natural reconstitution of CD8 lymphocytes occurred within 10 days and a much slower recovery of CD4 cells (Dudley et al. 2002a, b). This nonmyeloablative regimen also resulted in white blood cell and platelet decreases that also recovered by about 10 days. At the time of maximal lymphodepletion, cells were administered to 13 patients and 6 (46%) of these patients had objective clinical responses and 4 others demonstrated mixed responses. Five patients demonstrated signs of melanocyte destruction including four patients with vitiligo and one patient with anterior uveitis. Importantly, persistence of the transferred cells was dramatically increased and two of the patients appeared to undergo clonal repopulation of the transferred cells (63% and 97% of circulating CD8 cells were from the infusion bag). The use of this lymphodepleting regimen represented an important step in improving ACT and has become a mainstay of the preparation of patients for adoptive cell transfer (Dudley et al. 2003). Elegant experiment in mouse models in Nick Restifo’s laboratory defined many of the mechanisms surrounding the effectiveness of lymphodepletion. We then evaluated increased lymphodepletion by adding whole body irradiation to the chemotherapy regimen in a series of prospective pilot trials. Objective responses were seen in 49% in patients who received the lymphodepleting chemotherapy alone and was 52% and 72%, respectively, in patients who received added 2 Gy or 12 Gy irradiation (Dudley et al. 2005, 2008). The apparent increase in response rates using the 12 Gy regimen later led us to perform a prospective randomized trial in 101 patients, half of whom received TIL alone and half with 12 Gy whole body radiation, and there was no difference seen in response rates or survival in this trial (Goff et al. 2016). This was a further lesson that nonrandomized pilot trials can provide unreliable results. We published an analysis of the pilot trials in 2011 in 93 patients with metastatic melanoma (Rosenberg et al. 2011). Twenty of these patients (22%), achieved a complete tumor regression and 19 had ongoing complete regressions beyond 3 years. An analysis of these patients, performed predominantly by Paul Robbins, revealed several factors that were associated with objective response that included longer telomeres of the infused cells, the number of relatively undifferentiated CD8^+^ CD27^+^ cells infused and the persistence of the infused cells in the circulation at 1 month (all p2 < 0.001). These factors strongly suggested that less differentiated cells with higher proliferative capacity were the cells mediating the anti-tumor response, and in conjunction with similar findings in animal models being performed by Dr. Nicholas Restifo’s lab resulted in substantial attempts to improve the administration of cells with these properties in subsequent trials.

A current analysis of 192 consecutive melanoma patients treated with TIL grown from melanoma fragments and expanded to large amounts and reinfused into patients resulted in a 56% objective response rate in 192 checkpoint naïve patients including 48 patients (25%) with complete regressions and 9 patients (5%) with partial regression lasting beyond 5 years. Of the 48 patients who underwent complete regression only two have ever recurred with median potential follow up beyond 7 years and only two patients required a second treatment. The response rate to ACT using TIL in 34 patients who had previously received anti-PD1 checkpoint inhibitor therapy and who progressed resulted in a drop in the objective response rate to 24%; the reasons to explain this decrease are now under study.

The availability of T cell populations with specific recognition of melanomas enabled us to use these cells to attempt to clone human tumor antigens using expression cloning techniques. This laborious technique, first successfully used by Thierry Boon in Belgium to identify cancer germline antigens, was then utilized to identify a variety of both shared and mutated antigens recognized by T cells in patients with melanoma and other solid cancers (Bruggen et al. 1991).

Although melanoma seems to be susceptible to adoptive cell therapy utilizing unselected cells grown from resected melanoma tumor deposits, this approach did not result in anti-tumor responses in other histologies (except rarely in HPV induced (Stevanovic et al. 2015) cancers) and stimulated extensive efforts to identify whether or not solid epithelial cancers that represent about 90% of all cancer deaths in the United States, actually did express cancer antigens recognized by human T cells.

Cancer regressions mediated by TIL in melanoma patients rarely involved destruction of normal cells. This clue as well as the small number of mutated antigens had been discovered by cDNA expression cloning techniques led us to hypothesize that cancer mutations were the most likely targets of effective TIL and led us to develop in 2013 a new high-through-put screening approach to identify the potential immunogenicity of the products of cancer mutations in cancer cells (Lu et al. 2014; Robbins et al. 2013). The new screening approach involved whole exome sequencing of tumors to identify all mutated genes expressed in patient cancers. We then evaluated all mutated T cell epitopes by expressing them on the patient’s antigen presenting cells. This assay had multiple advantages because all non-synonymous cancer mutations presented on all of the patient’s MHC molecules were interrogated in a single assay. In updated studies of 76 patients with metastatic melanoma, 180 immunogenic epitopes were identified representing 1.3% of all mutations. Unexpectedly all of the neoantigens recognized were unique and none were shared among patients. In 75 patients with a variety of metastatic gastrointestinal cancers, 62 (83%) contained T-cells that recognized a total of 124 cancer mutations and all but one (KRAS G12D) were unique (Parkhurst et al. 2019). These studies have now been performed in 195 patients with common epithelial cancers. Approximately 80% of patients with all cancer histologies contain T cells capable of recognizing unique cancer mutations presented and expressed in their cancer. Of the 363 total neoantigens recognized in these 195 patients, 362 of the antigens were unique to the individual patient and only 2 patients recognized the same shared KRAS mutation recognized on the same Cw*0802 MHC restriction element. It thus appears that the overwhelming majority of human cancers are immunogenic and that they can give rise to T cells recognizing these unique cancer determinants.

In recent studies we are applying this information to identify cells that are uniquely reactive against the products of cancer mutations, and we are using highly selected TIL for the treatment of patients with these common epithelial cancers. The first patient to be treated with a population of highly selected cells recognizing a unique cancer mutation (ERBB2IP) in a patient with a cholangiocarcinoma resulted in a dramatic durable tumor regression (Tran et al. 2014). We have subsequently reported tumor regressions in patients with colon cancer, (Tran et al. 2016) and breast cancer (Zacharakis et al. 2018) showing that it is indeed possible to use adoptive cell therapy targeting unique cancer antigens to mediate tumor responses in a variety of cancer types.

These studies suggest that the recognition of random somatic mutations is the “final common” pathway explaining cancer regressions for most immunotherapies for solid cancers (Tran et al. 2017). Further it appears that any intracellular protein can potentially be a cancer antigen if mutated and processed intracellularly to a peptide that can bind to the autologous MHC. This is both good and bad news. Most cancer patients are potentially eligible for this kind of treatment because virtually all cancers contain mutations. Further, this treatment must be highly individualized since individual patients must be treated with T cells that recognize unique mutations in their cancer. Treatments thus will be highly complex. These studies are making inroads into the greatest problem that now exists in oncology, the development of cancer treatments for patients with metastatic solid epithelial cancers that result in 90% of cancer deaths around the world. As I write this review in 2021, that challenge is occupying all of my research efforts.

## Use of genetic modification of human lymphocytes to improve anti-tumor activity of adoptive cell therapy of cancer in patients

As the above studies of naturally occurring anti-tumor lymphocytes were proceeding, we were studying ways to genetically modify lymphocytes to improve their anti-tumor effectiveness. These studies were initially limited by the inefficient transfection of genes into human lymphocytes. We and others intensified our efforts to improve the development of retroviral vectors that could be used to insert these genes. In our group, these initial studies were led by Richard Morgan, who joined my laboratory in 2001 and Dr. Patrick Hwu who came to my lab in 1990 as part of his medical oncology training at the National Cancer Institute.

Tumor necrosis factor (TNF) had been described as a highly active anti-cancer molecule in mouse models and appeared to compromise the vasculature of rapidly growing tumors. We first attempted to use autologous TIL as vehicles to deliver TNF at high concentrations to a tumor deposit. The TNF gene was transduced into lymphocytes and the secreted levels of TNF were 30-fold lower than comparably transduced tumor cell lines transduced with this gene (Hwu et al. 1993a). Although secretion of tumor necrosis factor was low, we attempted this treatment in several patients, and in 1992, we treated a 52-year old woman with metastatic melanoma who had progressed after treatment using non-transduced TIL followed by multiple doses of IL-2 to keep these cells alive in vivo (Rosenberg 1992). We grew TIL from one of many subcutaneous lesions that she had throughout her body, transduced the lymphocytes with the gene encoding TNF, and injected escalating doses of these TNF gene modified TIL twice a week giving increasing doses of these cells in the absence of IL-2 administration. Multiple melanoma nodules regressed and ultimately disappeared, and the patient survived disease-free for several decades. The hemodynamic effects of tumor necrosis factor were of concern although there was no toxicity in these patients likely due to the very low amounts of TNF that were being produced. It was not at all clear that the TNF had played a role in the tumor reduction since some patients could potentially respond to a second treatment with naturally occurring TIL. The ability to successfully treat patients with this kind of functional modification of the T cell was the stimulus to proceed with efforts to genetically modify lymphocytes to improve anti-tumor activity.

At about this time, I heard a lecture by Dr. Zelig Eshhar, at the Weizman Institute in Israel who had developed a technique to alter lymphocyte function by constructing chimeric antigen receptors (CARs) that contained the variable regions of the heavy and light chain of antibodies connected by a flexible linker and joined to the constant region of the T cell receptor (Eshhar et al. 1993). Excited by the application of this potential technology to treat cancer, I quickly invited Dr. Eshhar to come to my laboratory at the National Cancer Institute on a sabbatical from the Weizmann Institute so we could work to apply this approach to cancer treatment in patients.

If we could utilize monoclonal antibodies recognizing cancer antigens to produce CARs and insert them into lymphocytes, we could potentially create tumor reactive lymphocytes free of the limitations of MHC restriction that is a characteristic of conventional T cell receptors. Dr. Eshhar came to my laboratory in 1990 and worked side by side with Patrick Hwu and other members of the lab. We started by constructing CARs targeting the 38-kD folate binding protein (FBP), a molecule overexpressed in most human ovarian cancers. Patrick Hwu showed that these CAR modified TIL could successfully be redirected to specifically target FBP in in vitro experiments (Hwu et al. 1993a, b). Nude mice that were given intraperitoneal implants of human ovarian cancer cells and were treated with anti FBP CAR transduced lymphocytes had increased survival compared to untreated mice (Hwu et al. 1995). In a second model mice with lung tumors induced by IV injection of methacholanthrene-induced sarcomas transduced with FBP could be treated by the systemic injection of these CAR expressing cells. A pilot clinical trial was then performed in sixteen patients with advanced cancer. These early studies demonstrated that it was possible to treat patients with these CAR T cells although no anti-tumor responses were seen. Dr. Michael Kershaw who worked with Patrick Hwu published this experience in 2006, and this served to stimulate interest in the use of CARs for anti-cancer treatment (Kershaw et al. 2006).

A major problem with the use of CARs for the treatment of solid cancers is the lack of monoclonal antibodies that could selectively recognize cancer and not normal cells. Our experience with CARs and TCRs had taught us that normal cells could be destroyed as quickly as cancer cells when attacked by lymphocytes, and this could lead to severe toxicity and death in patients. We painfully learned that lesson by treating a patient with T-cells targeting normal ERBB2 (Morgan et al. 2010) and in two patients with TCRs targeting MAGE-A3 (Morgan et al. 2013). These treatment related deaths were traumatic to me and the entire team. It reminded me of the experience of operating on a patient and having them die on the operating table. There is no doubt as to who is responsible for the tragedy.

As this work with CAR T cells was underway, we increasingly emphasized studies of the use of conventional alpha/beta T cell receptors from TIL that could recognize intracellular cancer antigens that were presented as peptides on cell surface MHC molecules. Using expression cloning techniques Yutaka Kawakami used our TIL from clinical trials of melanoma patients to identify two melanoma/melanocyte differentiation antigens called MART-1 and gp100 (Kawakami et al. 1994a, b).

The gene encoding a TCR that recognized the MART-1 antigen was isolated from a melanoma patient TIL obtained from a patient who demonstrated a near complete regression of metastatic melanoma after TIL transfer. We performed a pilot clinical trial in 15 patients with metastatic melanoma using peripheral blood lymphocytes transduced with a gene encoding the MART-1 reactive TCR (Morgan et al. 2006). Durable engraftment was seen at levels exceeding 10% of peripheral blood T-cell for at least 2 months after the infusion and, in some patients, engineered cells were found in the circulation a year later. Two patients demonstrated a sustained objective regression of their metastatic melanoma including one patient who experienced complete regression of an axillary mass and an 89% reduction of a liver metastatic deposit lasting 21 months after treatment. A second patient underwent a complete regression of a hilar mass and remained clinically disease-free over 10 years later. This clinical trial represented the first example of the ability of cells genetically modified to express T cells receptors to mediate cancer regression in humans (Morgan et al. 2006). Laura Johnson extended these studies by identifying a very highly avid T cell receptor targeting MART-1 which we then used to treat 20 patients with metastatic melanoma and an additional 16 patients who received autologous lymphocytes transduced with a mouse T cell receptor that recognized gp100 developed in Nicholas Restifo’s lab (Johnson et al. 2009). Objective response rates in these two trials were 30% and 19%, respectively. However, using these highly avid T cell receptors, severe auditory and ocular toxicity was seen in some patients likely due to the presence of melanocytes in the eye and the ear that were also targeted. By stopping treatment and administering local steroids, patients returned to their normal baseline function with no residual defects, but these toxicities led us to discontinue clinical trials targeting these normal melanocyte antigens. These studies were then extended by Paul Robbins in the Surgery Branch demonstrating that T cell receptors that targeted the NYESO-1 cancer germline antigen could also mediate tumor regression in patients (Robbins et al. 2015). As described in the prior section, we can now identify cancer antigens and their cognate T-cell receptors in most patients with common epithelial cancers and we are attempting to introduce those receptors into autologous lymphocytes for use in treatment.

These early studies demonstrated that genetically modified lymphocytes could mediate tumor regression and played a formative role in the development of modern gene therapy for hematologic and solid cancers.

Within months of our work utilizing T cell receptor transduction to treat solid tumors, we returned to work utilizing chimeric antigen receptors targeting the CD19 molecule present on most malignant B cells as well as mature B cells and B cell precursors. Earlier experiments by Michael Sadelain’s laboratory at Memorial Sloan Kettering demonstrated that lymphocytes expressing anti CD19 CARs could kill CD19 + primary leukemia cells in vitro and eliminate CD19 target cells in murine xenograft models (Brentjens et al. 2003). In addition data suggesting that adding co-stimulation moieties such as CD28 or 41BB to CARs could enhance antigen specific cytokine production and proliferation (Sadelain et al. 2013). These studies of CD19 as a target led to interest in using this cell transfer approach to treat patients bearing B cell malignancies.

**Fig. 9:**
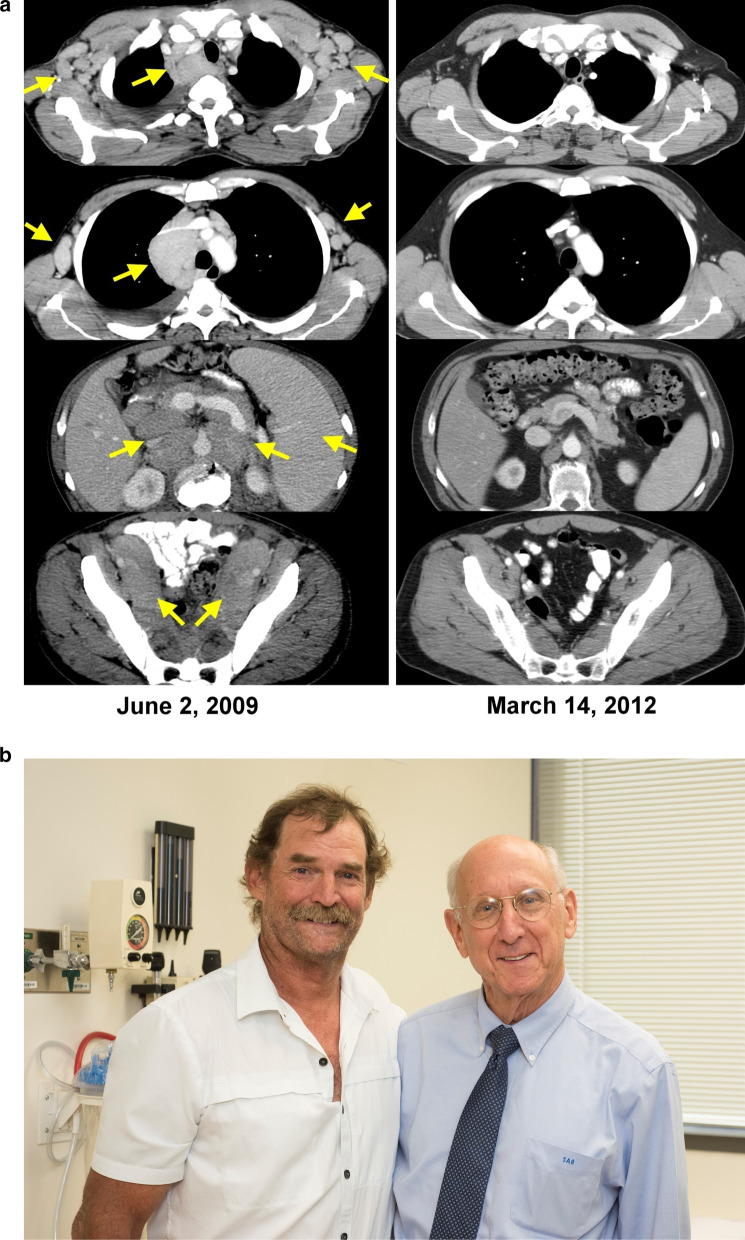
**a** CAT Scans of the first patient to respond to treatment (in 2009) with adoptive transfer of autologous lymphocytes genetically engineered to express a chimeric antigen receptor targeting CD19 (left, lymphoma burden shown with arrows); right, complete cancer regression now ongoing over 10 years later. **b** In clinic with the first patient 10 years after treatment

In March 2007, James Kochenderfer, a medical oncology/hematology fellow in the National Cancer Institute, approached me about leveraging our ongoing work on T cell receptor transfer and CAR T cell transfer in order to target CD 19 for the treatment of patients with advanced lymphomas. Jim joined my lab and constructed two CARs that contained a mouse anti-human CD19 antibody chain derived from the FMC63 mouse hybridoma. These retroviral vectors encoded a single chain antibody and a portion of the CD28 costimulatory molecule as well as the cytoplasmic component of the TCR zeta chain molecule in the MSGV retroviral backbone. He selected a CAR showing the greatest potency in vitro when transduced into CD8 + and CD4 + T cells and showed highly specific activity against CD19 expressing tumor cells as measured by lysis and cytokine production. In a syngeneic mouse lymphoma model the infusion of anti-CD19 CAR T-cells could eradicate tumor if preceded by a lymphodepleting whole body irradiation (Kochenderfer et al. 2010a). In May 2009, following extensive in vitro and murine studies, we treated the first patient with autologous peripheral lymphocytes transduced with our anti-CD19 CAR construct (Kochenderfer et al. 2010b). This first patient was a 48-year-old male who had a Non-Hodgkin Lymphoma and had previously been treated with four different regimens including aggressive combination chemotherapies. The patient had extensive bulky disease in lymph nodes, mediastinum and abdomen, and following two cycles of CD19 CAR T-cell treatment, he experienced a complete regression which is ongoing now over 10 years later (Figs. 9a, b). This report published in 2010 and our ongoing studies showing objective responses in six of the first seven patients treated, including five complete responses, stimulated considerable interest in this approach (Kochenderfer et al. 2010b, 2011). Ten months after our first publication the group at the University of Pennsylvania reported anti-tumor responses using anti-CD-19 T cells to treat three children with acute lymphocytic leukemia (Porter et al. 2011). Our group and others went on to treat large numbers of patients that confirmed the ability of this approach to mediate regressions in patients with advanced diffuse large B cell lymphomas and acute lymphocytic leukemia (Kochenderfer et al. 2017).

As we were reporting these dramatic responses to anti CD-19 CAR T cell therapy, I was contacted in 2011 by Arie Belldegrun, a former fellow, then a professor of Urology at UCLA, who had worked in my lab 25 years earlier. Arie had a vision of how to commercialize this approach for the treatment of hematologic cancers. In 2012 the Surgery Branch NCI signed a Cooperative Research and Development Agreement (CRADA) with Kite Pharma founded by Dr. Belldegrun. We transferred our technology to Kite and worked closely with them to develop a closed system for cell production, applicable to Good Manufacturing Practices. Clinical studies in the Surgery Branch confirmed by subsequent multi institutional studies by Kite Pharma resulted in objective responses in about 83% of patients with diffuse, large B cell lymphoma with 58% complete durable responses (Cappell et al. 2020; Neelapu et al. 2017). Both Kite Pharma and Novartis, who had been conducting trials in conjunction with the University of Pennsylvania group, received FDA approval to market CD19 CAR for the treatment of diffuse large B cell lymphoma and acute lymphocytic leukemia, respectively in 2017. In that same month, Kite was sold to Gilead Sciences for 11.9 billion dollars. Anti-CD19 CAR studies are now being widely used in patients throughout the United States, Europe, and Israel. Over 200 companies world-wide are now working to develop cell-based therapies.

## Concluding remarks

When I came to the National Cancer Institute on July 1, 1974 as Chief of the Surgery Branch (a position I have held continuously for the past 46 years) little if anything was known about the possible relationships between the immune system and growing cancers in humans. Much has been learned in the succeeding four decades by many research groups especially in the area of cell transfer therapy and checkpoint modulators (CPM) inhibiting CTLA-4 and PD-1. Although we worked briefly on CPM and reported the first human clinical responses to CPM administration (Phan et al. 2003) using anti-CTLA-4 in 2003, the seminal background development of this approach and its extensive clinical application were performed by others (Sharma and Allison 2015) and I have not discussed this subject in this monograph. In this brief review, I have attempted to give a sense of the start and progress of efforts in my laboratory at the National Cancer Institute to develop effective cell-based immunotherapies for patients with cancer.

In the first 10 years at the NCI, I performed laboratory and small animal studies of immune reactions to cancer though we made no progress in successfully applying these findings to the treatment of patients with cancer. During this time we treated 76 patients using the transfer of lymphokine activated killer (LAK) cells with non-specific tumor reactivity or the administration of mammalian and later recombinant interleukin-2 as a non-specific immune stimulator and saw no clinical responses in these patients. It was only in 1985, using the infusion of recombinant interleukin-2 at high doses and with an appropriate schedule based on pharmacokinetic studies, that we treated a patient with metastatic melanoma who underwent complete disappearance of her disease (now ongoing over 36 years later). Other responses quickly followed, and we went on to treat over 600 cancer patients with IL-2, results that played an important role in its ultimate approval as the first cancer immunotherapy approved by the FDA. These studies with IL-2 showed for the first time that an immunologic maneuver could mediate cancer regression in humans.

Our work proceeded to explain the mechanism of these IL-2 mediated responses and led in 1988 to the demonstration that tumor infiltrating lymphocytes (TIL) obtained from the stroma of a growing tumor, expanded in vitro in IL-2 and administered to patients could mediate tumor regression. This work ultimately led to objective response rates of 56% including 22% with durable complete remissions in patients with metastatic melanoma. These evolving results were the basis of our efforts over the next 30 years to use lymphocytes as a “living drug” for cancer treatment.

We performed a cascade of studies utilizing T cell transfer including the development of genetically modified lymphocytes to provide them with properties to improve their reactivity against cancer and these studies ultimately led to our ability to transduce chimeric antigen receptors (CARs) targeting CD19 into autologous lymphocytes for the treatment of patients with aggressive lymphomas, the first cell therapy for cancer approved by the FDA.

In the course of this work, we investigated many approaches that did not work in patients and that led to substantial delays in progress. Our studies of non-specific lymphokine activated killer (LAK) cells intrigued us with its impact on small mouse tumors but its lack of effectiveness in a prospective randomized trial led us to ultimately abandon its use. In retrospect, we pursued it longer than we should have. We learned a great deal from our early studies targeting normal melanoma/melanocyte antigens but also painfully learned that targeting these normal non-mutated antigens including Her-2, CEA and MAGE-A3 could lead to the destruction of normal tissues and severe toxicities in patients, including death. We developed assays to determine the molecular nature of the antigens recognized by human T cells that ultimately led us to the demonstration that immune reactivity against the products of cancer mutations was likely the “final common pathway” of most if not all immunotherapy for the treatment of cancer patients and thus avoided reactivity to normal tissues.

In the course of our studies of cell transfer immunotherapy, we spent considerable time attempting to develop therapeutic cancer vaccines. We treated 428 patients with a variety of cancer vaccines using shared as well as unique tumor antigens without success (Rosenberg et al. 2004) and to the present time there have been no direct immunization strategies that have been effective in cancer treatment.

The major challenge now confronting cancer immunotherapy is the development of effective treatments for patients with metastatic solid epithelial cancers that result in 90% of all cancer deaths. In the past 5 to 6 years I have devoted our efforts solely to that goal. We have demonstrated that about 80% of all patients with common epithelial cancers contain T cells that recognize the products of unique mutations in the autologous cancer and that targeting those mutations can sometimes lead to cancer regression. We now have seen responses in selected patients with a variety of common cancers such as colon, breast, and cervical cancer and current efforts are working to further refine this approach.

## References

[CR1] Atkins MB, Lotze MT, Dutcher JP, Fisher RI, Weiss G, Margolin K (1999). High-dose recombinant interleukin-2 therapy for patients with metastatic melanoma: analysis of 270 patients treated between 1985 and 1993. J Clin Oncol.

[CR2] Brentjens RJ, Latouche JB, Santos E, Marti F, Gong MC, Lyddane C (2003). Eradication of systemic B-cell tumors by genetically targeted human T lymphocytes co-stimulated by CD80 and interleukin-15. Nat Med.

[CR3] Cappell KM, Sherry RM, Yang JC, Goff SL, Vanasse DA, McIntyre L (2020). Long-term follow-up of anti-CD19 chimeric antigen receptor T-cell therapy. J Clin Oncol.

[CR4] Cheever MA, Greenberg PD, Fefer A (1981). Specific adoptive therapy of established leukemia with syngeneic lymphocytes sequentially immunized in vivo and in vitro and nonspecifically expanded by culture with interleukin 2. J Immunol.

[CR5] Donohue JH, Rosenberg SA (1983). The fate of interleukin-2 after in vivo administration. J Immunol.

[CR6] Donohue JH, Lotze MT, Robb RJ, Rosenstein M, Braziel RM, Jaffe ES (1984). In vivo administration of purified Jurkat-derived interleukin-2 in mice. Cancer Res.

[CR7] Dudley ME, Wunderlich JR, Robbins PF, Yang JC, Hwu P, Schwartzentruber DJ (2002). Cancer regression and autoimmunity in patients after clonal repopulation with anti-tumor lymphocytes. Science.

[CR8] Dudley M, Wunderlich J, Yang JC, Hwu P, Schwartzentruber DJ, Topalian SL (2002). A phase I study of non-myeloablative chemotherapy and adoptive transfer of autologous tumor antigen-specific T lymphocytes in patients with metastatic melanoma. J Immunother.

[CR9] Dudley ME, Wunderlich JR, Shelton TE, Even J, Rosenberg SA (2003). Generation of tumor-infiltrating lymphocyte cultures for use in adoptive transfer therapy for melanoma patients. J Immunother.

[CR10] Dudley ME, Wunderlich JR, Yang JC, Sherry RM, Topalian SL, Restifo NP (2005). Adoptive cell transfer therapy following non-myeloablative but lymphodepleting chemotherapy for the treatment of patients with refractory metastatic melanoma. J Clin Oncol.

[CR11] Dudley ME, Yang JC, Sherry R, Hughes MS, Royal R, Kammula U (2008). Adoptive cell therapy for patients with metastatic melanoma: evaluation of intensive myeloablative chemoradiation preparative regimens. J Clin Oncol.

[CR12] Eberlein TJ, Rosenstein M, Rosenberg SA (1982). Regression of a disseminated syngeneic solid tumor by systemic transfer of lymphoid cells expanded in IL-2. J Exp Med.

[CR13] Eshhar Z, Waks T, Gross G, Schindler DG (1993). Specific activation and targeting of cytotoxic lymphocytes through chimeric single chains consisting of antibody-binding domains and the gamma or zeta subunits of the immunoglobulin and T-cell receptors. Proc Natl Acad Sci.

[CR14] Fisher B, Packard BS, Read EJ, Carrasquillo JA, Carter CS, Topalian SL (1989). Tumor localization of adoptively transferred Indium-111 labeled tumor infiltrating lymphocytes in patients with metastatic melanoma. J Clin Oncol.

[CR15] Goff SL, Dudley ME, Citrin DE, Somerville RP, Wunderlich JR, Danforth DN (2016). Randomized, prospective evaluation comparing intensity of lymphodepletion before adoptive transfer of tumor-infiltrating lymphocytes for patients with metastatic melanoma. J Clin Oncol.

[CR16] Greenberg PD, Cheever MA, Fefer A (1981). Eradication of disseminated murine leukemia by chemoimmunotherapy with cyclophosphamide and adoptively transferred immune syngeneic Lyt-1+2- lymphocytes. J Exp Med.

[CR17] Griffith KD, Read EJ, Carrasquillo JA, Carter CS, Yang JC, Fisher B (1989). In vivo distribution of adoptively transferred indium-111 labeled tumor infiltrating lymphocytes and peripheral blood lymphocytes in patients with metastatic melanoma. J Natl Cancer Inst.

[CR18] Grimm EA, Mazumder A, Zhang HZ, Rosenberg SA (1982). The lymphokine activated killer cell phenomenon: lysis of natural killer-resistant fresh solid tumor cells by interleukin 2-activated autologous human peripheral blood lymphocytes. J Exp Med.

[CR19] Hewitt HB, Blake ER, Walder AS (1976). A critique of the evidence for active host defence against cancer, based on personal studies of 27 murine tumours of spontaneous origin. Br J Cancer.

[CR20] Hwu P, Yannelli J, Kriegler M, Anderson WF, Perez C, Chiang Y (1993). Functional and molecular characterization of tumor infiltrating lymphocytes transduced with the tumor necrosis factor-a cDNA for the gene therapy of cancer in man. J Immunol.

[CR21] Hwu P, Shafer GE, Treisman J, Schindler DG, Gross G, Cowherd R (1993). Lysis of ovarian cancer cells by human lymphocytes redirected with a chimeric gene composed of an antibody variable region and the Fc receptor gamma chain. J Exp Med.

[CR22] Hwu P, Yang JC, Cowherd R, Treisman J, Shafer GE, Rosenberg SA (1995). In vivo antitumor activity of T-cells redirected with chimeric antibody/T-cell receptor genes. Cancer Res.

[CR23] Johnson LA, Morgan RA, Dudley ME, Cassard L, Yang JC, Hughes MS (2009). Gene therapy with human and mouse T-cell receptors mediates cancer regression and targets normal tissues expressing cognate antigen. Blood.

[CR24] Kammula US, White DE, Rosenberg SA (1998). Trends in the safety high dose bolus interleukin-2 administration in patients with metastatic cancer. Cancer.

[CR201] Kawakami Y, Eilyahu S, Delgado CH, Robbins PF, Sakaguchi K, Appella E, Yannelli JR, Adema GJ, Miki T, Rosenberg SA. Identification of a human melanoma antigen recognized by tumor-infiltrating lymphocytes associated with in vivo tumor rejection. Proc Natl Acad Sci. 1994a;91:6458–62. 10.1073/pnas.91.14.6458 PMC44221 8022805

[CR202] Kawakami Y, Eliyahu S, Delgado CH, Robbins PF, Rivoltini L, Topalian SL, Miki T, and Rosenberg SA. Cloning of the gene coding for a shared human melanoma antigen recognized by autologous T cells infiltrating into tumor. Proc Natl Acad Sci. 1994b;91:3515–19. 10.1073/pnas.91.9.3515 PMC43610 8170938

[CR25] Kershaw MH, Westwood JA, Parker LL, Wang G, Eshhar Z, Mavroukakis SA (2006). A phase I study on adoptive immunotherapy using gene-modified T cells for ovarian cancer. Clin Cancer Res.

[CR26] Kochenderfer JN, Yu Z, Frasheri D, Restifo NP, Rosenberg SA (2010). Adoptive transfer of syngeneic T cells transduced with a chimeric antigen receptor that recognizes murine CD19 can eradicate lymphoma and normal B cells. Blood.

[CR27] Kochenderfer JN, Wilson WH, Janik JE, Dudley ME, Stetler-Stevenson M, Feldman SA (2010). Eradication of B-lineage cells and regression of lymphoma in a patient treated with autologous T cells genetically engineered to recognize CD19. Blood.

[CR28] Kochenderfer JN, Dudley ME, Feldman SA, Wilson WH, Spaner DE, Maric I (2011). B-cell depletion and remissions of malignancy along with cytokine-associated toxicity in a clinical trial of anti-CD19 chimeric-antigen-receptor-transduced T cells. Blood.

[CR29] Kochenderfer JN, Somerville RPT, Lu T, Yang JC, Sherry RM, Feldman SA (2017). Long-duration complete remissions of diffuse large B cell lymphoma after anti-CD19 chimeric antigen receptor T cell therapy. Mol Ther.

[CR30] Lotze MT, Strausser JL, Rosenberg SA (1980). In vitro growth of cytotoxic human lymphocytes. II. Use of T cell growth factor (TCGF) to clone human T cells. J Immunol.

[CR31] Lotze MT, Line BR, Mathisen DJ, Rosenberg SA (1980). The in vivo distribution of autologous human and murine lymphoid cells grown in T cell growth factor (TCGF): Implications for the adoptive immunotherapy of tumors. J Immunol.

[CR32] Lotze MT, Grimm EA, Mazumder A, Strausser JL, Rosenberg SA (1981). Lysis of fresh and cultured autologous tumor by human lymphocytes cultured in T cell growth factor (TCGF). Cancer Res.

[CR33] Lotze MT, Frana LW, Sharrow SO, Robb RJ, Rosenberg SA (1985). In vivo administration of purified human interleukin-2. I. Half life and immunologic effects of the Jurkat cell line derived interleukin 2. J Immunol.

[CR34] Lu YC, Yao X, Crystal JS, Li YF, El-Gamil M, Gross C (2014). Efficient identification of mutated cancer antigens recognized by T cells associated with durable tumor regressions. Clin Cancer Res.

[CR35] Morgan DA, Ruscetti FW, Gallo R (1976). Selective in vitro growth of T lymphocytes from normal human bone marrows. Science.

[CR36] Morgan RA, Dudley ME, Wunderlich JR, Hughes MS, Yang JC, Sherry RM (2006). Cancer regression in patients after transfer of genetically engineered lymphocytes. Science.

[CR37] Morgan RA, Yang JC, Kitano M, Dudley ME, Laurencot CM, Rosenberg SA (2010). Case report of a serious adverse event following the administration of T cells transduced with a chimeric antigen receptor recognizing ERBB2. Mol Ther.

[CR38] Morgan RA, Chinnasamy N, Abate-Daga D, Gros A, Robbins PF, Zheng Z (2013). Cancer regression and neurological toxicity following anti-MAGE-A3 TCR gene therapy. J Immunother.

[CR39] Mule JJ, Shu S, Schwarz SL, Rosenberg SA (1984). Adoptive immunotherapy of established pulmonary metastases with LAK cells and recombinant interleukin-2. Science.

[CR40] Mule JJ, Shu S, Rosenberg SA (1985). The anti-tumor efficacy of lymphokine-activated killer cells and recombinant interleukin-2 in vivo. J Immunol.

[CR41] Mule JJ, Yang J, Shu S, Rosenberg SA (1986). The anti-tumor efficacy of lymphokine-activated killer cells and recombinant interleukin-2 in vivo: direct correlation between reduction of established metastases and cytolytic activity of lymphokine-activated killer cells. J Immunol.

[CR42] Muul LM, Spiess PJ, Director EP, Rosenberg SA (1987). Identification of specific cytolytic immune responses against autologous tumor in humans bearing malignant melanoma. J Immunol.

[CR43] Neelapu SS, Locke FL, Bartlett NL, Lekakis LJ, Miklos DB, Jacobson CA (2017). Axicabtagene ciloleucel CAR T-cell therapy in refractory large B-cell lymphoma. N Engl J Med.

[CR44] Parkhurst MR, Robbins PF, Tran E, Prickett TD, Gartner JJ, Jia L (2019). Unique neoantigens arise from somatic mutations in patients with gastrointestinal cancers. Cancer Discov.

[CR45] Phan GQ, Yang JC, Sherry RM, Hwu P, Topalian SL, Schwartzentruber DJ (2003). Cancer regression and autoimmunity induced by cytotoxic T lymphocyte-associated antigen 4 blockade in patients with metastatic melanoma. Proc Natl Acad Sci.

[CR46] Porter DL, Levine BL, Kalos M, Bagg A, June CH (2011). Chimeric antigen receptor-modified T cells in chronic lymphoid leukemia. N Engl J Med.

[CR47] Robb RJ, Kutny RM, Chowdhry V (1983). Purification and partial sequence analysis of human T-cell growth factor. Proc Natl Acad Sci U S A.

[CR48] Robbins PF, Lu YC, El-Gamil M, Li YF, Gross C, Gartner J (2013). Mining exomic sequencing data to identify mutated antigens recognized by adoptively transferred tumor-reactive T cells. Nat Med.

[CR49] Robbins PF, Kassim SH, Tran TL, Crystal JS, Morgan RA, Feldman SA (2015). A pilot trial using lymphocytes genetically engineered with an NY-ESO-1 reactive T-cell receptor: long-term follow-up and correlates with response. Clin Cancer Res.

[CR50] Rosenberg SA (1992). Gene therapy for cancer. J Am Med Assoc.

[CR51] Rosenberg SA, Einstein AB (1972). Sialic acids on the plasma membrane of cultured human lymphoid cells: chemical aspects and bio-synthesis. J Cell Biol.

[CR52] Rosenberg SA, Guidotti G (1968). The protein of human erythrocyte membranes. I. Preparation, solubilization and partial characterization. J Biol Chem.

[CR53] Rosenberg SA, Guidotti G (1969). The fractionation of the protein components of human erythyrocyte membranes. J Biol Chem.

[CR54] Rosenberg SA, Fox E, Churchill WH (1972). Spontaneous regression of hepatic metastases from gastric carcinoma. Cancer.

[CR55] Rosenberg SA, Spiess PJ, Schwarz S (1978). In vitro growth of murine T-cells. I. Production of factors necessary for T-cell growth. J Immunol.

[CR56] Rosenberg SA, Schwarz S, Spiess PJ (1978). In vitro growth of murine T-cells. II. Growth of in vitro sensitized cells cytotoxic for alloantigens. J Immunol.

[CR57] Rosenberg SA, Schwarz S, Spiess PJ, Brown JM (1980). In vitro growth of murine T cells. III. Method for separation of T cell growth factor (TCGF) from concanavalin A and biological activity of the resulting TCGF. J Immunol Methods.

[CR58] Rosenberg SA, Spiess PJ, Schwarz S (1980). In vitro growth of murine T cells. IV. Use of T cell growth factor (TCGF) to clone lymphoid cells. Cell Immunol.

[CR59] Rosenberg SA, Grimm EA, McGrogan M, Doyle M, Kawasaki E, Koths K (1984). Biological activity of recombinant human interleukin-2 produced in *E. coli*. Science.

[CR60] Rosenberg SA, Mule JJ, Spiess PJ, Reichert CM, Schwarz S (1985). Regression of established pulmonary metastases and subcutaneous tumor mediated by the systemic administration of high dose recombinant IL-2. J Exp Med.

[CR61] Rosenberg SA, Lotze MT, Muul LM, Leitman S, Chang AE, Ettinghausen SE (1985). Observations on the systemic administration of autologous lymphokine-activated killer cells and recombinant interleukin-2 to patients with metastatic cancer. N Engl J Med.

[CR62] Rosenberg SA, Spiess P, Lafreniere R (1986). A new approach to the adoptive immunotherapy of cancer with tumor-infiltrating lymphocytes. Science.

[CR63] Rosenberg SA, Packard BS, Aebersold PM, Solomon D, Topalian SL, Toy ST (1988). Use of tumor-infiltrating lymphocytes and interleukin-2 in the immunotherapy of patients with metastatic melanoma. A preliminary report. N Engl J Med.

[CR64] Rosenberg SA, Aebersold PM, Cornetta K, Kasid A, Morgan RA, Moen R (1990). Gene transfer into humans: Immunotherapy of patients with advanced melanoma, using tumor-infiltrating lymphocytes modified by retroviral gene transduction. N Engl J Med.

[CR65] Rosenberg SA, Lotze MT, Yang JC, Topalian SL, Chang AE, Schwartzentruber DJ (1993). Prospective randomized trial of high-dose interleukin-2 alone or in conjunction with lymphokine-activated killer cells for the treatment of patients with advanced cancer. JNCI.

[CR66] Rosenberg SA, Yang JC, Topalian SL, Schwartzentruber DJ, Weber JS, Parkinson DR (1994). Treatment of 283 consecutive patients with metastatic melanoma or renal cell cancer using high-dose bolus interleukin-2. JAMA.

[CR67] Rosenberg SA, Yannelli JR, Yang JC, Topalian SL, Schwartzentruber DJ, Weber JS (1994). Treatment of patients with metastatic melanoma using autologous tumor-infiltrating lymphocytes and interleukin-2. J Natl Cancer Inst.

[CR68] Rosenberg SA, Yang JC, Schwarztentruber DJ, Hwu P, Marincola FM, Topalian SL (1999). Prospective randomized trial of the treatment of patients with metastatic melanoma using chemotherapy with cisplatin, dacarbazine, and tamoxifen alone or in combination with interleukin-2 and alpha-interferon. J Clin Oncol.

[CR69] Rosenberg SA, Yang JC, Restifo NP (2004). Cancer immunotherapy: moving beyond current vaccines. Nat Med.

[CR70] Rosenberg SA, Yang JC, Sherry RM, Kammula US, Hughes MS, Phan GQ (2011). Durable complete responses in heavily pretreated patients with metastatic melanoma using T-cell transfer immunotherapy. Clin Cancer Res.

[CR71] Rosenstein M, Eberlein T, Kemeny MM, Sugarbaker PH, Rosenberg SA (1981). In vitro growth of murine T cells: VI. Accelerated skin graft rejection caused by adoptively transferred cells expanded in T cell growth factor. J Immunol.

[CR72] Sadelain M, Brentjens R, Riviere I (2013). The basic principles of chimeric antigen receptor design. Cancer Discov.

[CR73] Sharma P, Allison JP (2015). The future of immune checkpoint therapy. Science.

[CR74] Smith FO, Downey SG, Klapper JA, Yang JC, Sherry RM, Royal RE (2008). Treatment of metastatic melanoma using interleukin-2 alone or in conjunction with vaccines. Clin Cancer Res.

[CR75] Spiess PJ, Rosenberg SA (1981). A simplified method for the production of murine T cell growth factor free of lectin. J Immunol Methods.

[CR76] Stevanovic S, Draper LM, Langhan MM, Campbell TE, Kwong ML, Wunderlich JR (2015). Complete regression of metastatic cervical cancer after treatment with human papillomavirus-targeted tumor-infiltrating T cells. J Clin Oncol.

[CR77] Strausser JL, Rosenberg SA (1978). In vitro growth of cytotoxic human lymphocytes. I. Growth of cells sensitized in vitro to alloantigens. J Immunol.

[CR78] Symes MO, Riddell AG, Feneley RC, Tribe CR (1973). The treatment of advanced bladder cancer with sensitized pig lymphocytes. Br J Cancer Suppl.

[CR79] Taniguchi T, Matsui H, Fujita T, Takaoka C, Kashima N, Yoshimoto R (1983). Structure and expression of a cloned cDNA for human interleukin-2. Nature.

[CR80] Topalian S, Solomon D, Avis FP, Chang AE, Freerksen DL, Linehan WM (1988). Immunotherapy of patients with advanced cancer using tumor infiltrating lymphocytes and recombinant interleukin-2: a pilot study. J Clin Oncol.

[CR81] Topalian SL, Solomon D, Rosenberg SA (1989). Tumor-specific cytolysis by lymphocytes infiltrating human melanomas. J Immunol.

[CR82] Tran E, Turcotte S, Gros A, Robbins PF, Lu Y-C, Dudley M (2014). Cancer immunotherapy based on mutation-specific CD4+ T cells in a patient with epithelial cancer. Science.

[CR83] Tran E, Robbins PF, Lu YC, Prickett TD, Gartner JJ, Jia L (2016). T-cell transfer therapy targeting mutant KRAS in Cancer. N Engl J Med.

[CR84] Tran E, Robbins PF, Rosenberg SA (2017). 'Final common pathway' of human cancer immunotherapy: targeting random somatic mutations. Nat Immunol.

[CR85] Van der Bruggen P, Traversari C, Chomez P, Lurquin C, DePlaen E, Van Den Eynde B (1991). A gene encoding an antigen recognized by cytolytic T lymphocytes on a human melanoma. Science.

[CR86] Wang A, Lu S-D, Mark DF (1984). Site-specific mutagenesis of the human interleukin-2 gene: structure-function analysis of the cyteine residues. Science.

[CR87] Yang JC, Mule JJ, Rosenberg SA (1985). Characterization of the murine lymphokine-activated killer precursor and effector cell. Surg Forum.

[CR88] Yang JC, Sherry RM, Steinberg SM, Topalian SL, Schwartzentruber DJ, Hwu P (2003). Randomized study of high-dose and low-dose interleukin-2 in patients with metastatic renal cancer. J Clin Oncol.

[CR89] Yron I, Wood TA, Spiess PJ, Rosenberg SA (1980). In vitro growth of murine T cells: V. The isolation and growth of lymphoid cells infiltrating syngeneic solid tumors. J Immunol.

[CR90] Zacharakis N, Chinnasamy H, Black M, Xu H, Lu YC, Zheng Z (2018). Immune recognition of somatic mutations leading to complete durable regression in metastatic breast cancer. Nat Med.

