# Correction to: A Journey in Science: Immersion in the search for effective cancer immunotherapies

**DOI:** 10.1186/s10020-021-00405-0

**Published:** 2021-11-02

**Authors:** Steven A. Rosenberg

**Affiliations:** National Cancer Institute, National Institutes of Health, Bethesda, MD USA

## Correction to: Mol Med (2021) 27:63 https://doi.org/10.1186/s10020-021-00321-3

Following publication of the original article (Rosenberg et al. 2021), the editorial team requested to change the article title as the author Steven A. Rosenberg received the journal Cerami award.

The incorrect title is: Immersion in the search for effective cancer immunotherapies

The correct title is: A Journey in Science: Immersion in the search for effective cancer immunotherapies

We have also added the below Abstract to introduce Dr. Rosenberg’s submission in context of the Cerami award.

Abstract

Real innovations in medicine and science are historic and singular; the stories behind each occurrence are precious. At Molecular Medicine we have established the Anthony Cerami Award in Translational Medicine to document and preserve these histories. The monographs recount the seminal events as told in the voice of the original investigators who provided the crucial early insight. These essays capture the essence of discovery, chronicling the birth of ideas that created new fields of research and launched trajectories that persisted and ultimately influenced how disease is prevented, diagnosed, and treated. In this volume, the Cerami Award Monograph is by Steven A. Rosenberg, Chief of Surgery at the National Cancer Institute in Bethesda, Maryland, USA. A pioneer in the development of immunotherapies and gene therapies for advanced cancers, this is the story of Dr. Rosenberg’s scientific journey.

The original article has been corrected.
